# Hoxc13 and homologs of mammalian hair keratins are required for the cornification of nuptial pads in *Xenopus* frogs

**DOI:** 10.1038/s42003-026-10519-y

**Published:** 2026-06-20

**Authors:** Julia Steinbinder, Attila Placido Sachslehner, Bahar Golabi, Marjolein Carron, Kris Vleminckx, Leopold Eckhart

**Affiliations:** 1 Department of Dermatology, Medical University of Vienna, Vienna, Austria; 2 Department of Biomedical Molecular Biology, Ghent University, Ghent, Belgium

**Keywords:** Evolutionary developmental biology, Differentiation

## Abstract

The evolution of skin appendages has involved innovation at the levels of keratin intermediate filament proteins, which build stable cornified structures, and regulatory factors, which determine the anatomical location of skin appendages. The keratin components of nails and claws have evolved at the digit tips under the control of the transcription factor Hoxc13 in a common ancestor of tetrapods. The additional expression of *hoxc13* at other skin sites allowed the evolution of hair in mammals. Here, we show that the cornification of epithelial spines within nuptial pads of *Xenopus* frogs involves the expression of the type II hair keratin homolog, *krt59*. The expression of *hoxc13* is enriched in the skin of the forelimbs, and CRISPR-Cas9-mediated inactivation of *hoxc13* abolished both the expression of *krt59* and epithelial cornification in nuptial pads of *X. tropicalis*. We conclude that cornified nuptial spines have evolved in parallel to mammalian hair by adaptation of the ancestral cornification program of skin appendages.

## Abstract (web summary)

*Xenopus* frogs depend on the evolutionarily conserved transcription factor Hoxc13 and homologs of hair keratins to form nuptial pads, suggesting that the genetic programs for cornified skin appendages are similar in amphibians and mammals.

## Introduction

Skin appendages, such as claws, hair, and feathers, evolved when vertebrates adapted to different environments and lifestyles after the transition from fully aquatic to terrestrial life^1–3^. The skin epithelium and the mesenchyme contribute to the development of skin appendages^3,4^. However, the mature, mechanically resilient appendages consist only of dead and interconnected epithelial cells that are packed with structural proteins such as keratins^5,6^. As several skin appendages are defining traits of major clades of vertebrates, e.g., hair in mammals and feathers in birds, the evolution of skin appendages is an important research paradigm^1,7–9^. Studies of skin appendages in a broad range of species have revealed examples of conservation of ancient features and innovation of lineage-specific traits as drivers of skin appendage evolution^10–13^. Amphibians are phylogenetically basal tetrapods, and research into cornified skin appendages of amphibians appears to be essential for understanding the early evolution of skin appendages, besides informing about specific adaptations to the amphibian lifestyles.

Nuptial pads are one of the few types of cornified skin appendages, besides claws and larval beaks, which are found in amphibians (frogs and toads) and urodeles (salamanders)^14–19^. Nuptial pads are arrays of microscopic spines, also called hillocks^20^ or hooks^21^, that develop on the inner aspects of forelimbs, including the toes of sexually mature anurans, to support the amplexus during mating^15^. In line with their function, nuptial pads are generally found in male individuals. However, nuptial pad-like structures develop on the forelimbs of females in some species, such as the African clawed frog (*Xenopus laevis*) and the Torres redbelly toad (*Melanophryniscus macrogranulosus*)^14,22^. In *X. laevis*, nuptial pads typically cover the entire ventral surface of the forelimbs of male frogs, whereas nuptial pad-like structures are restricted to the ventral surfaces of the digits of female frogs. The latter contain fewer but larger cornified spines^14^. Nuptial pads of frogs are evolutionarily related to the nuptial pads of newts^16^ and the nuptial spines on the maxillary skin of male moustache toads^23,24^. Histologically, the nuptial pads are continuous with the neighboring epidermis, and like in the epidermis^25^, epithelial cells, known as keratinocytes, differentiate by accumulating keratin intermediate filaments and eventually form a cornified layer (stratum corneum)^14,20,26,27^. Langerhans cells and dendritic cells are sparsely distributed between epithelial cells of the amphibian epidermis^25^.

Hair keratins are keratin intermediate filament proteins which are specifically expressed in hair and other cornified skin appendages of tetrapods. Human hair keratins are classified into the monophyletic clades of type I (KRT31-KRT40) and type II (KRT81-KRT86) hair keratins^28–30^, which undergo heterodimerization. Hair keratins are the main constituents of hair, nails, claws, horns, and filiform papillae of the tongue^30,31^. Orthologs of mammalian hair keratins have been identified in reptiles^7^ and amphibians^24,32,33^. In *Xenopus* frogs, the hair keratin homologs Krt34 (type I) and Krt59 (type II) are expressed in the cornified claws^13^, which is equivalent to the co-expression of type I and type II hair keratins in the claws of anole lizards^7^. The conserved expression of hair keratin homologs in the claws of mammals, lizards, and frogs has suggested that the evolutionarily primordial function of these keratins was the protection of toe tips and the mechanical stabilization of projecting claws^13^.

The expression of hair keratins is regulated by the transcription factor Hoxc13^34^. In humans, mutations in *HOXC13* are linked to ectodermal dysplasia 9, characterized by defects in hair and nail morphogenesis^35,36^. Similarly, *Hoxc13* loss-of-function mutations result in impaired development of hair and nails in mice, rabbits, and pigs^37–39^. In the chicken, *HOXC13* is expressed in developing claws^9^. Recently, we have generated *hoxc13*-deficient *X. tropicalis* frogs, in which the expression of hair keratin homologs on the toe tips and the development of claws were suppressed, indicating that the regulatory role of Hoxc13 in the cornified claws has evolved in a common ancestor of amniotes and amphibians^13^.

In the present study, we investigated the roles of hair keratin homologs and Hoxc13 in nuptial pads of *Xenopus* frogs. We show that the cornification of epithelial spines within nuptial pads shares molecular features with the cornification of *Xenopus* claws. Importantly, our study extends the characterization of the molecular evolution of skin appendages in amphibians and reveals a key role of Hoxc13 in the evolutionary innovation of phylogenetically diverse cornified skin appendages.

## Results

### Nuptial pads of *Xenopus* frogs contain cornified skin appendages and display unique gene transcription profiles

**Fig. 1:**
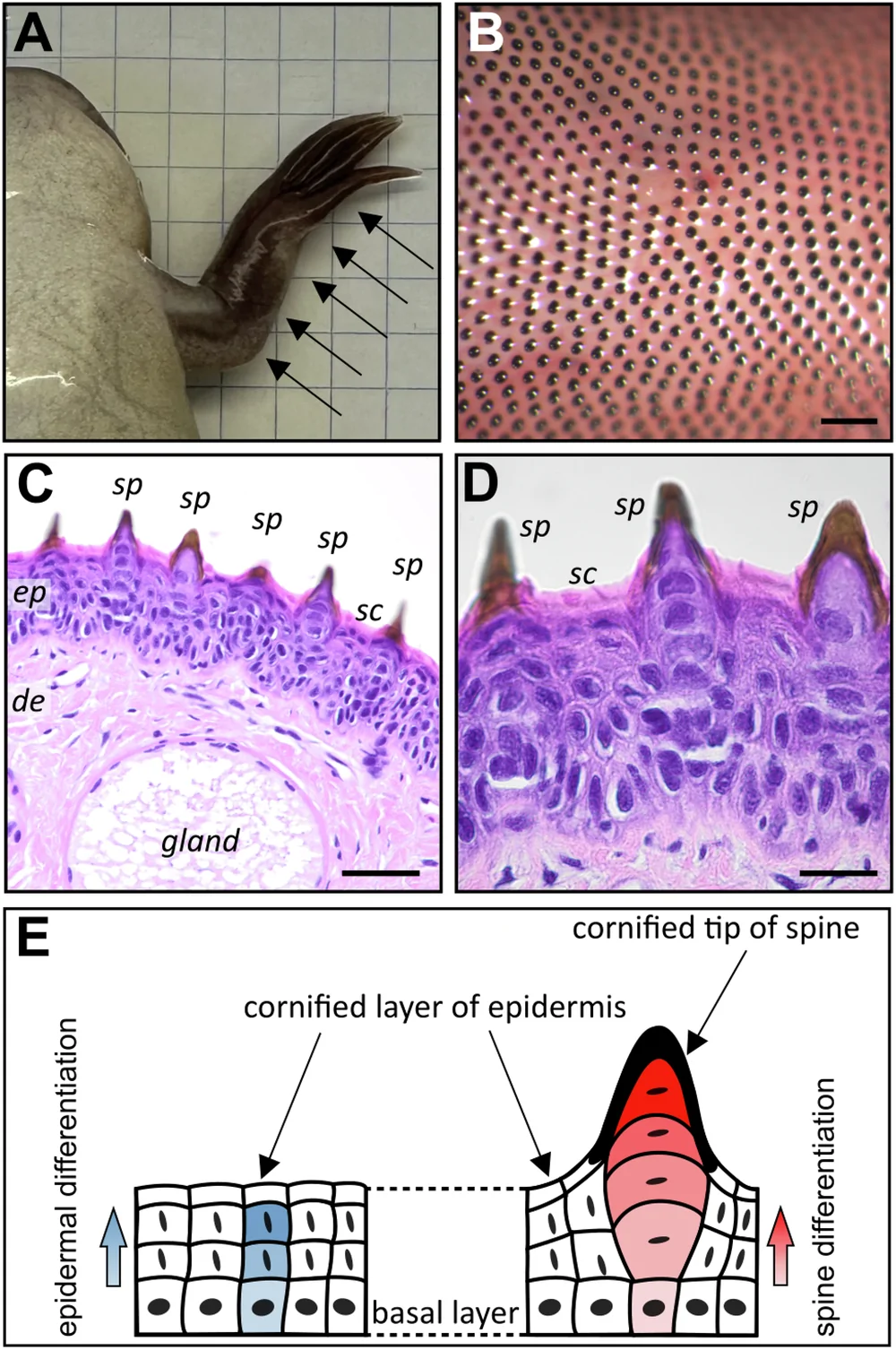
Nuptial pads of *Xenopus* frogs contain cornified epithelial spines. **A** Macroscopic image of a forelimb of male *X. laevis* frog at an age of 5 years. Arrows point to nuptial pads. The side length of the squares in the background is 5 mm. **B** Stereomicroscopic image of male *X. laevis* nuptial pad. **C**, **D** Hematoxylin and eosin staining of nuptial pads of male *X. laevis* frogs. Spines (sp) are indicated. Images at a higher magnification reveal a regular organization of epithelial cells in the spines, which are separated by epidermis covered by a stratum corneum (sc) in male frogs. **E** Schematic depiction of keratinocyte differentiation in nuptial spines *versus* epidermis. ep epidermis, de dermis, sp spine, sc stratum corneum. Scale bars: 100 µm (**B**), 50 µm (**C**), and 20 µm (**D**).

The cornified spines of *X. laevis* frogs were examined at the macroscopic and the microscopic level. The ventral surface of the forelimbs and forelimb toes of male frogs (Fig. 1A–C) and the ventral side of the forelimb toes of female frogs (Supplementary Fig. S1) were covered by regularly spaced, brown skin projections. In both male (Fig. 1D) and female (Supplementary Fig. S1) frogs, each spine consisted of a pile of epidermal cells with enucleate brown cells forming the tips. In agreement with a previous report^14^, individual spines were larger but less densely arranged in female frogs (Supplementary Fig. S2).

The cornified cells at the tip of the spines were clearly distinct from the flat cornified cell layer between the spines within the nuptial pads of *Xenopus* frogs (Fig. 1D). Cornified spines were absent from the skin covering other body sites, such as the upper portion of the hindlimbs (Supplementary Fig. S3), from where samples were taken for subsequent comparative analyses. We concluded that the epithelial spines of nuptial pads are cornified skin appendages that form by a cell differentiation program distinct from the conventional program of cell differentiation in the epidermis (Fig. 1E).

**Fig. 2:**
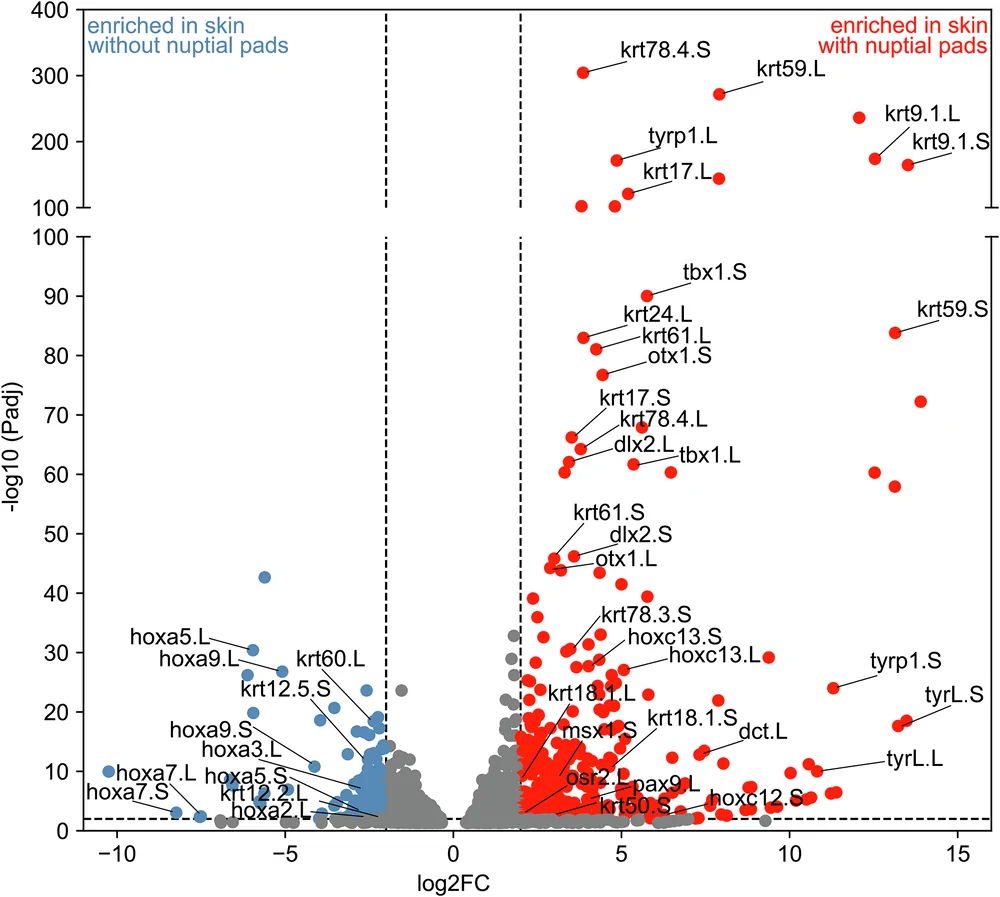
Transcriptomics reveals the unique gene expression profile of nuptial pads. Volcano plot displaying differentially expressed genes between *X. laevis* forelimb skin with nuptial pads (*n* = 3) and hindlimb skin (*n* = 3) as a control. Each point represents a transcript, plotted by log2FC (horizontal axis) and −log10 (*P*adj) (vertical axis). Genes significantly upregulated in skin with nuptial pads are highlighted in red, while those enriched in control skin without nuptial pads are shown in blue, non-significant genes are depicted in gray. Selected marker genes with known roles in keratinization, pigmentation, and signaling are annotated. The data underlying the volcano plot, including log2FC and −log10(*P*adj) values for all transcripts, are provided in Supplementary Data S3. FC fold change, *P*adj, adjusted *P* value.

To identify the differences in gene expression programs of skin with nuptial pads (forelimbs) and skin without nuptial pads (hindlimbs), we performed RNA sequencing and subsequent differential gene expression analysis. In total, 339 genes were significantly upregulated in forelimb skin containing nuptial pads (log2FC > 2, *P*adj<0.01), whereas 162 genes were enriched in control skin on the hindlimbs of male *X. laevis* frogs (Fig. 2 and Supplementary Data S1). Keratins, which are specifically expressed in epithelial cells^5^, and homeobox transcription factors were prominent among the differentially expressed genes (Fig. 2).

### Type II hair keratin homologs are expressed in the cornified spines of nuptial pads

Among keratins, the type II hair keratin homologs *krt59.L* and *krt59.S* were highly elevated in nuptial pads as compared to control skin (Fig. 2 and Table 1). The type I keratins *krt9.1.L* and *krt9.1.S* were enriched with similar fold-changes as *krt59.L* and *krt59.S* in nuptial pad tissue, whereas the type I hair keratin homologs *krt34.L* and *krt34.S* were not expressed. Proteomic analysis of nuptial pads confirmed that the expression of Krt59.L and Krt59.S at the protein level and the absence of Krt34 homeologs (Supplementary Data S2). This pattern suggests that the conventional heterodimerization of type I and type II hair keratin homologs, as it occurs in *Xenopus* claws^13^, is substituted by the dimerization of type II hair keratin homologs with type I non-hair keratins. Notably, *krt59* and *krt9* homologs were top-ranked with regard to differential expression relative to skin without nuptial pads, but—in line with the large portion of inter-spine epithelium and undifferentiated epithelium (Fig. 1D)—other keratins reached higher read counts in nuptial pads (Table 1). In addition to the afore-mentioned *krt59* and *krt9* homeologs, several uncharacterized keratins such as *krt17.L* were elevated with lower fold changes in nuptial pads, and others such as *krt12.5.S* were decreased in comparison to control skin (Table 1).

**Table 1:** Expression levels of keratin genes in skin containing nuptial pads and skin without nuptial pads

*FSK* forelimb skin, *HSK* hindlimb skin, *lfcSE* standard error of the estimated log2 fold change, *stat* Wald test statistic (*Z* value) with *n* = 3 biologically independent samples per tissue type was computed as the ratio of the estimated log2 fold change to its standard error, P*adj* adjusted *P* value.

Notes: Keratin genes that reached a normalized read count of more than 100 in at least one sample are included in this table. Color shading in the second to seventh column is directly proportional to the normalized read counts. Log2FC values are color-coded from red (enriched in nuptial pads) to blue (enriched in skin without nuptial pads).

**Fig. 3:**
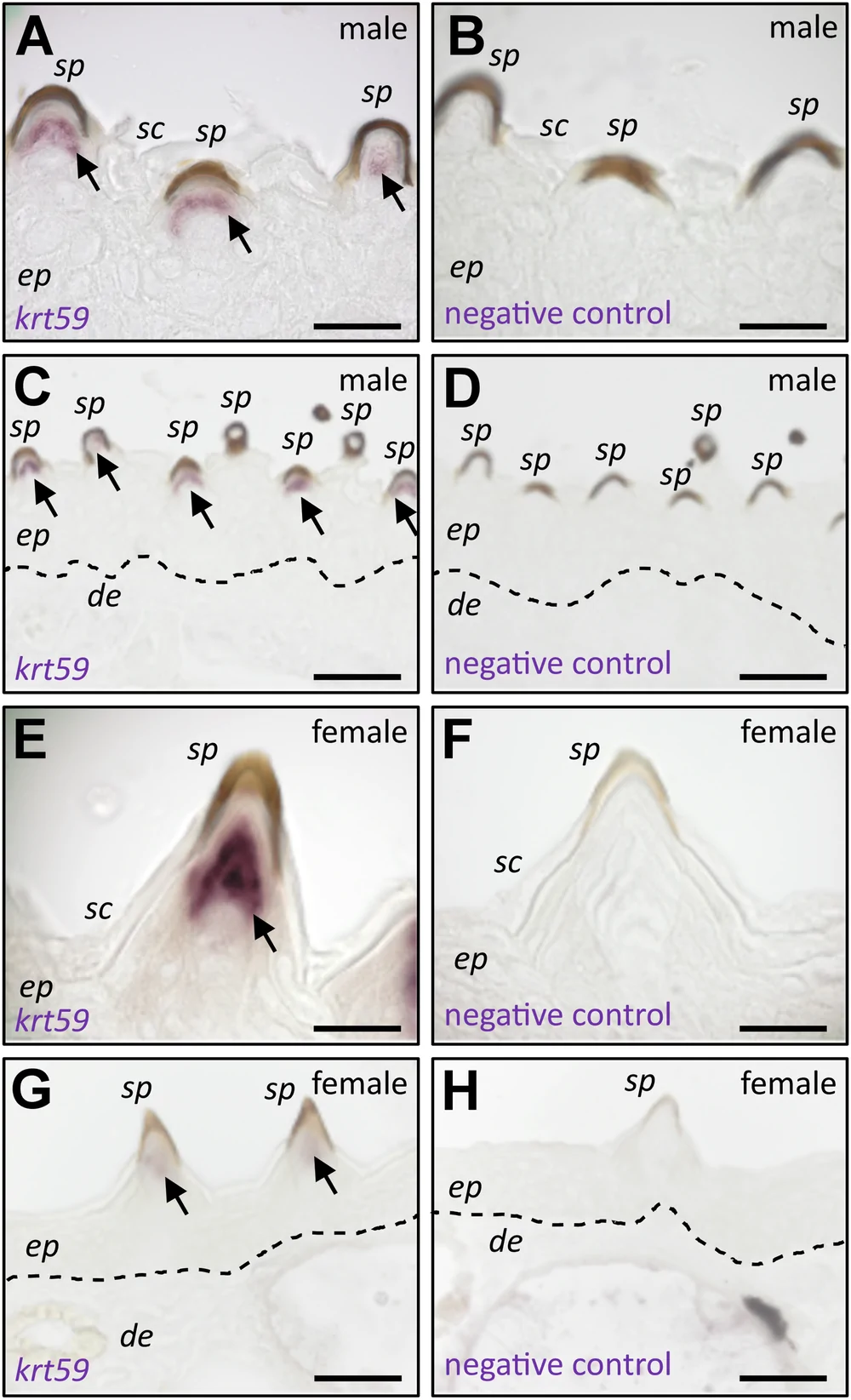
Hair keratin homolog *krt59* is expressed in cornified spines within nuptial pads and nuptial pad-like structures of *X. laevis*. *krt59* mRNAs were detected by in situ mRNA hybridization using an antisense probe for both *krt59.L* and *krt59.S* homeologs in sections of forelimb skin of male (**A**, **C**) and female (**E**, **G**) *X. laevis* frogs. In negative control experiments, a probe in sense orientation was used on serial sections (**B**, **D**, **F**, **H**). sp spine, ep epidermis, sc stratum corneum. Scale bars: 20 µm (**A**, **B**, **E**, **F**), 50 µm (**C**, **D**, **G**, **H**).

To localize the expression of *krt59*, mRNA in situ hybridization was performed on forelimb skin sections of *X. laevis* frogs. The mRNA of *krt59* was distinctly localized in the keratinocytes close to the tip of the cornified spines within nuptial pads of both male (Fig. 3A) and female frogs (Fig. 3C). *krt59* was not expressed in the epidermis between the spines and in the skin outside of the nuptial pads. No signal was detected using the negative control probe consisting of an RNA transcribed in sense orientation (Fig. 3B, D). These results demonstrate that *krt59* is specifically expressed in the differentiated epithelial cells of the nuptial pad, consistent with a role of Krt59 as a structural component of the cornified tip of the spines.

### Hoxc13 is expressed in nuptial pads and required for the expression of hair keratin homologs and cornification of epithelial spines in *Xenopus* nuptial pads

Among Hox genes, *hoxc12* and *hoxc13* were significantly elevated in forelimb skin with nuptial pads of *X. laevis*, whereas several members of the Hoxa family were enriched in hindlimb skin without nuptial pads (Table 2). In line with their location at the end of the *hoxc* gene cluster, *hoxc12* and *hoxc13* are expressed in posterior body parts during embryonic development of vertebrates, but they are also involved in the development and regeneration of limbs^40^, the differentiation of claws^13^, and the growth of mammalian hair^41^. Both homeologs of Hoxc13, *hoxc13.L* and *hoxc13.S*, were expressed at high levels as compared to all other hox genes in nuptial pads (Table 2).

**Table 2:** Hox genes differentially expressed in skin containing nuptial pads and skin without nuptial pads

*FSK* forelimb skin, *HSK* hindlimb skin, *log2FC* log2 fold change, *lfcSE* standard error of the estimated log2 fold change, *stat* Wald test statistic (*Z* value) with *n* = 3 biologically independent samples per tissue type was computed as the ratio of the estimated log2 fold change to its standard error, P*adj* adjusted *P* value.

Notes: Color shading in the second to seventh column is directly proportional to the normalized read counts. Log2FC values are color-coded from red (enriched in nuptial pads) to blue (enriched in skin without nuptial pads).

**Fig. 4:**
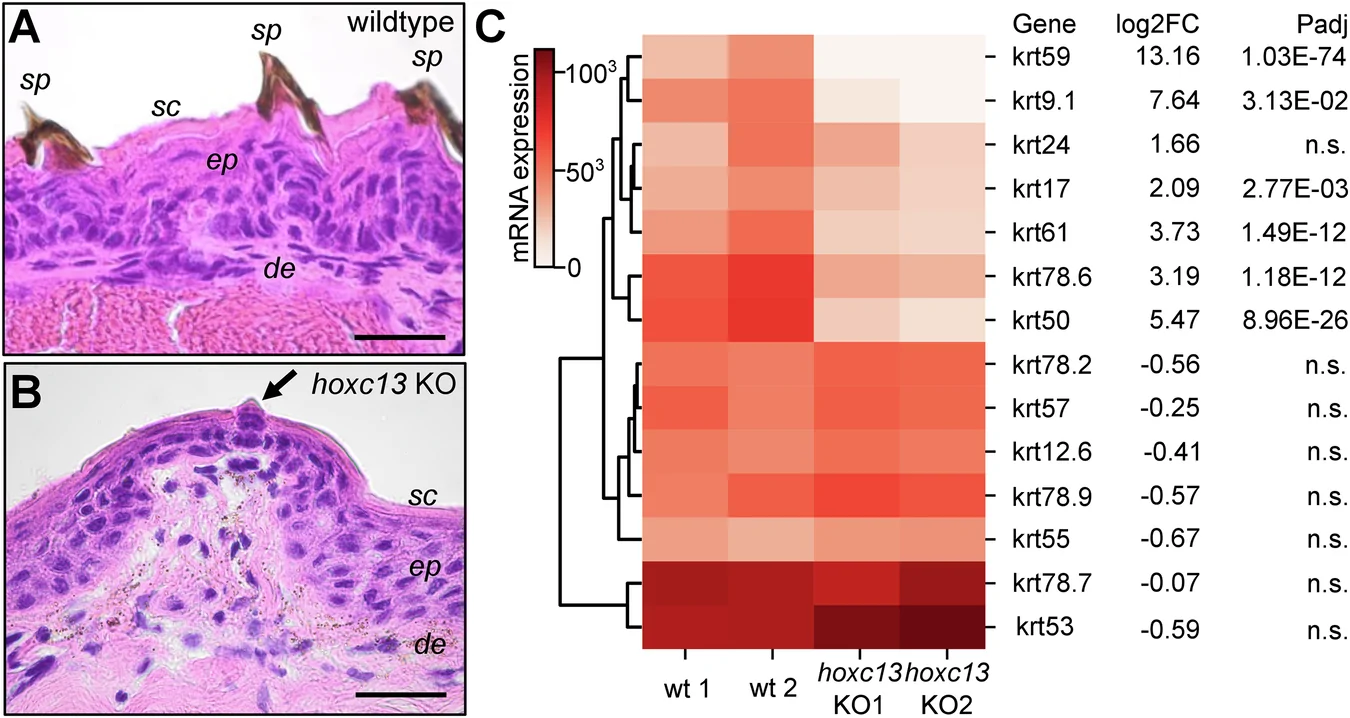
Phenotypic and transcriptomic differences in nuptial pad development between wild-type and Hoxc13-deficient *X. tropicalis*. Hematoxylin and eosin staining of forelimb toe skin from wild-type (**A**) and Hoxc13-deficient (*hoxc13* KO) (**B**) frogs. An arrow points to an epidermal projection lacking pigmentation and cornification. The images are representative of two male frogs at an age of 7 months per genotype. **C** Clustermap of RNA-seq expression values (normalized counts) of keratin genes in forelimb toes of wild-type (*n* = 2) *versus* *hoxc13* knockout (*n* = 2) frogs. Genes with >20,000 normalized read counts in wild-type (wt) tissue are included in the graph, in the corresponding Supplementary Table S1 and in the Supplementary Data S3 (source data of heatmap), whereas expression values of all keratin genes are shown in Supplementary Table S2. Samples are column-ordered by genotype (wt1, wt2, *hoxc13* KO1, *hoxc13* KO2). Genes (rows) are hierarchically clustered. Log2 fold-change (log2FC) values refer to the ratio of expression levels in wild-type *versus* Hoxc13 knockout. sp spine, ep epidermis, de dermis, n.s. not significant (*P*adj ≥ 0.05), *P*adj adjusted *P* value, sc stratum corneum. Scale bars: 20 µm.

To investigate whether Hoxc13 contributes to the formation and cornification of nuptial pads, we investigated samples from wild-type *X. tropicalis* frogs in comparison to Hoxc13-deficient frogs that had been generated in a previous study^13^. Histological examination revealed that the skin of the forelimb toes of wild-type frogs displayed cornified spines characteristic of nuptial pads (Fig. 4A). In contrast, Hoxc13-deficient frogs formed only rudimentary epidermal projections which lacked a pigmented cornified surface (Fig. 4B). The Hoxc13-deficient structures were smaller (height 12 µm *vs*. 23 µm) and less densely arranged (mean distance 0.7 spines/100 µm *vs*.1.9 spines/100 µm) than the epithelial spines of wild-type frogs.

To assess the molecular basis of this phenotype, RNA-sequencing was performed on forelimb toe skin from wild-type and *hoxc13* knockout *X. tropicalis* frogs (Supplementary Fig. S4). The hair keratin homologs *krt59*, *krt9.1*, and to a lesser extent, several other keratins were downregulated upon depletion of Hoxc13 (Fig. 4C and Supplementary Tables S1 and S2). Like in claw tissue^13^, the CRISPR/Cas9-mediated disruption of *hoxc13* did not abolish *hoxc13* mRNA in nuptial pads (Supplementary Table S3). Both in claws and nuptial pad sites of *hoxc13* mutant frogs, *hoxc12*, which is the immediate neighbor of *hoxc13*, was upregulated (Supplementary Tables S3 and S4). Taken together, these data demonstrate that Hoxc13 is essential for the activation of the keratinization program underlying nuptial pad cornification, supporting its conserved role as a master regulator of hair keratin-dependent cornification of vertebrate skin appendages.

## Discussion

This study shows that a specific epithelial differentiation program underlies the formation of the cornified spines of the nuptial pads in *Xenopus* frogs and demonstrates that this program depends on the transcription factor Hoxc13 and the type II hair keratin homolog Krt59. The involvement of these genes is conserved in nuptial pads and cornified claws of *Xenopus* frogs^13^, and Hoxc13 and hair keratins are also major determinants of cornified skin appendages in mammals. Accordingly, the results of this study have implications for the evolution of skin appendages in tetrapods.

In the course of the evolution of mammals, hair keratins were repurposed from ancestral roles in cornified claws to other cornified structures, such as hair shafts, filiform papillae on the tongue, and cornified scales on the tail of rodents^6,31^. All these mammalian cornified skin appendages have retained the expression of both type I and II hair keratins. Similarly, *Xenopus* claws contain homologs of type I and II hair keratins^13^. By contrast, the nuptial pads of *Xenopus* frogs lack expression of type I hair keratins. The absence of the type I hair keratin homolog (Krt34) from nuptial pads suggests that *Xenopus* Krt59 depends on an alternative dimerization partner. The strong upregulation of the *krt9.1* gene in nuptial pads indicates that the corresponding Krt9.1 protein is a potential heterodimerization partner for Krt59. Notably, *Xenopus krt9.1* is not a 1:1 ortholog of mammalian *KRT9*, which is predominantly expressed in palm and sole skin^42^ but phylogenetically equally close to several mammalian keratin genes^43^. A comparable lack of type I:type II hair keratin dimerization occurs in turtles and birds, which have a type II hair keratin homolog but not a type I hair keratin homolog^43,44^.

The enriched expression of *hoxc13* in nuptial pads aligns with its established roles in mammalian cornified skin appendages^37,45^ and claws of *Xenopus* frogs^13^. In *Hoxc13* knockout mice, defects impact multiple hard cornified structures, including hair, nails, and filiform papillae, supporting its regulatory influence in cornified tissues^37^. Similarly, the Hoxc13-dependent keratin defects observed in pigs and rabbits underscore the conserved function of this transcription factor across mammals^38,39^. Our findings indicate that this evolutionary conservation is not restricted to cornified claws^13^ but also occurs in other cornified structures of amphibians. Hoxc13-deficient *X. tropicalis* frogs lack expression of *krt59* and fail to form the pigmented, fully cornified tips of the nuptial pad spines; instead they develop epidermal projections lacking the dark terminal cornified layer (Fig. 4B). Thus, Hoxc13 is not only a quantitatively predominant Hox transcription factor in nuptial pads but also an essential regulator of the differentiation program in cornified spines within nuptial pads (Fig. 1E). However, the absence of expression of *krt34*, which like *krt59* contains a Hoxc13 binding site in its proximal promoter^13^, suggests that keratin expression is not controlled by Hoxc13 alone in nuptial pads. The reason for the difference between *krt34* and *krt59* expression patterns is currently unknown but may involve differential dependency on additional transcriptional activators besides Hoxc13, active repression of *krt34* in the nuptial pads, or the dependence of *krt34* on higher levels of Hoxc13 than those present in nuptial pad epithelial cells. Although abundant according RNA-seq analysis, *hoxc13* mRNA was not detectable by in situ hybridization in nuptial pads. By contrast, the claw epithelium was clearly positive in *hoxc13* in situ hybridization^13^, suggesting a lower level of expression of *hoxc13* in nuptial pads than in the claws.

Besides *krt59*, other members of the large family of keratin genes^46^ were also affected either directly or indirectly by the loss of Hoxc13 (Fig. 4C and Supplementary Tables S4 and S5). Several members of the Hox family and homologs of transcription factors implicated in mammalian skin and body appendages were altered in their expression levels upon loss of Hoxc13 (Supplementary Tables S4 and S5). Remarkably, many appendage-associated transcription factors were enriched in the skin with nuptial pads *versus* skin without nuptial pads (Supplementary Fig. S5 and Supplementary Table S6). Furthermore, the transcriptome of nuptial pads (this study) differed from that of claws^13^ (Supplementary Table S7). For example, the transcription factor Pax9, homologous to a mammalian regulator of hair keratin genes in filiform papillae of the tongue^47^ was enriched in nuptial pads but absent in claws. The links between Hoxc13 and other transcription factors, potentially including evolutionarily conserved regulators of skin appendages, will be important topics of future studies. While the present study focused on the cornified epithelial spines of nuptial pads, the abundance and differentiation of skin glands, which are also important for the function of nuptial pads^15^, remain to be investigated with regard to their keratin profile and their dependence on Hoxc13.

**Fig. 5:**
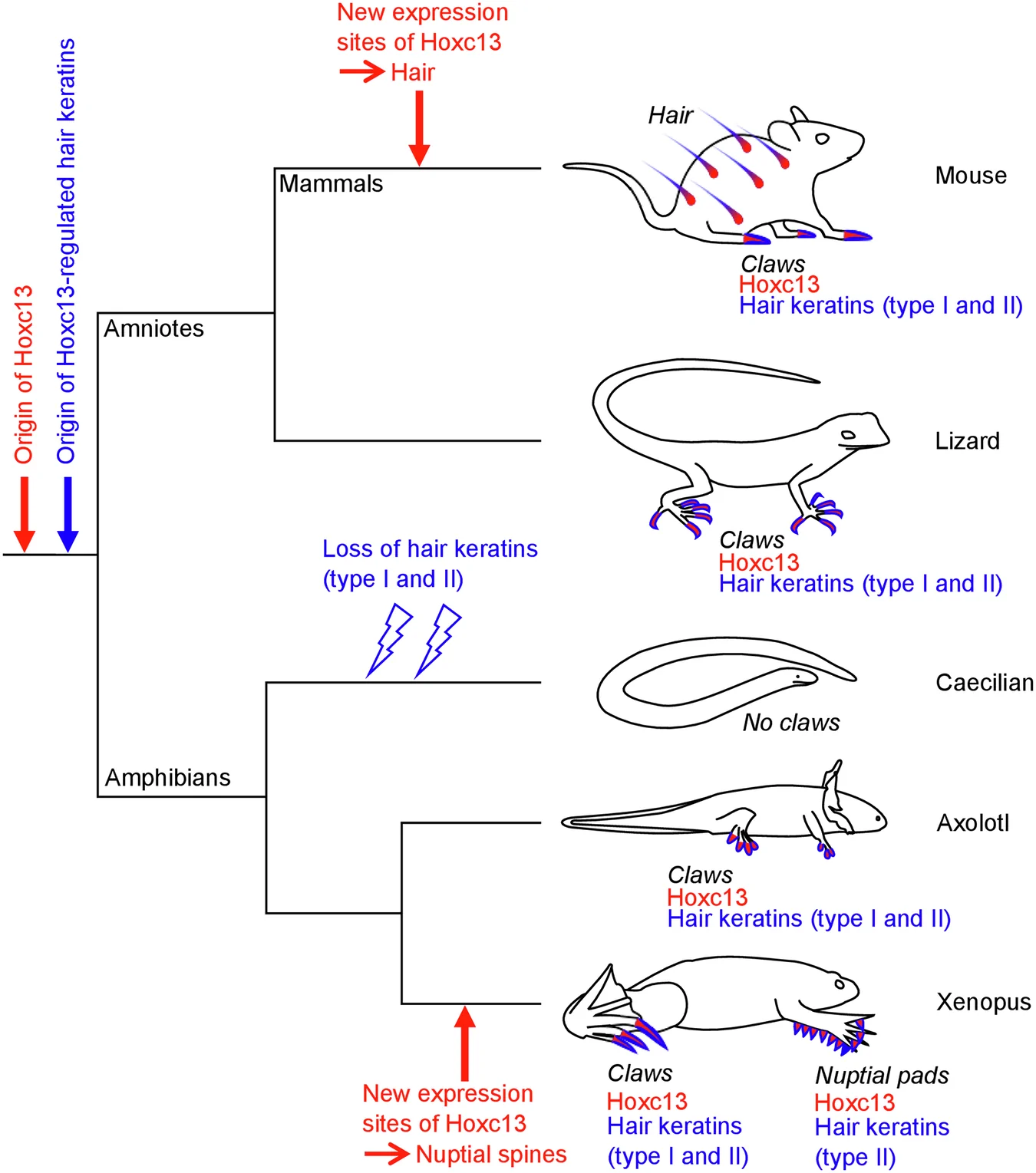
Evolutionary model of Hoxc13-dependent hard keratinization across tetrapods. The schematic diagram shows how Hoxc13-dependent hard keratinization has evolved outside claws in both mammals and amphibians. Sites of expression of Hoxc13 and hair keratins are highlighted in red and blue shading, respectively. Only the expression of Hoxc13 in adult animals is depicted, whereas the evolutionarily ancestral role of Hoxc13 in the development of the posterior part of the body is not included in the diagram. Hence, the indicated origin of Hoxc13 refers to the origin of Hoxc13-dependent regulation of skin appendages. “Hair keratins” refers to human keratins KRT31-KRT40 and KRT81-KRT86 and their orthologs in other species. Red arrows indicate evolutionarily new expression sites of Hoxc13, the blue arrow indicates the origin of hair keratins, and blue flash symbols indicate the loss of hair keratins.

In conclusion, our data provide evidence that Hoxc13-dependent differentiation of skin appendages is a conserved and versatile development program. In stem tetrapods, Hoxc13 was associated with epithelial differentiation on digit tips from which claws and nails evolved in modern tetrapods^13^. During the evolution of tetrapods, Hoxc13 was co-opted to skin appendages at other body sites, including hair, filiform papillae of the tongue, and tail scales in mammals^37^ and nuptial pads in amphibians. The evolutionary diversification of the Hoxc13-dependent differentiation program facilitated the recurrent origin of hard epidermal structures across vertebrate lineages. The model presented in Fig. 5 proposes that shifts in Hoxc13 expression, combined with gains or losses of hair keratin orthologs, provided the foundation for the emergence of novel skin appendages in both mammals and amphibians.

## Methods

### Animals tissue and ethics

*X. laevis* frogs were kindly provided by the Elly Tanaka Lab, Institute of Molecular Pathology, Vienna. The animals were killed solely for the purpose of obtaining their tissues and organs, and no procedures causing pain, suffering, or distress within the meaning of the Austrian Animal Experimentation Act (Tierversuchsgesetz 2012) were performed prior to euthanasia. No experiments on live animals were performed. The animals were killed by euthanization, and tissues were sampled according to the Animal Care and Use Committee of the Medical University of Vienna, and all experiments were conducted according to the guidelines established by the Animal Care and Use Committee of the Medical University of Vienna. *hoxc13* gene-edited *X. tropicalis* frogs (Supplementary Fig. S4) were generated in a previous study^13^. *hoxc13* knockout and wild-type frogs were kept under conditions reported previously^13^. All experiments involving *X. tropicalis* were conducted in compliance with the guidelines and regulations of Ghent University, Faculty of Sciences, Ghent, Belgium. The project was approved by the Ethical Committee for Animal Experimentation, Ghent University, Faculty of Sciences (approval number EC2023-041). We have complied with all relevant ethical regulations for animal use.

### Tissue sampling and fixation

For euthanasia, 5-year-old *X. laevis* frogs were exposed to a 1 g/L tricaine mesylate (MS-222) solution (catalog number E10521-10G, Sigma-Aldrich). Prior to tissue sampling, macroscopic images of the frogs were captured perpendicularly with a camera integrated in a mobile phone (iPhone 14 Pro Max, Apple). In addition, forelimbs were examined and images were captured using a Leica MZ16 A stereoscope with an Leica CLS 150 X external light source equipped with a Leica IC 3D camera and the software Leica Application Suite (LAS, version: 4.7.1). Tissue for RNA extraction was dissected and placed in RNAlater (catalog number R0901, Sigma-Aldrich), while tissue intended for histological analysis was stored in 7.5% neutral-buffered formalin (NBF) (catalog number FN-1000-75-1, SAV Liquid Production GmbH) overnight. Toes previously fixed in NBF were subsequently decalcified by incubating them for 3 days in a solution composed of 5% formic acid (catalog number 695076, Honeywell-Fluka) and 0.2 M EDTA, adjusted to pH 8.0 with sodium hydroxide. Then the samples were thoroughly washed with water and incubated in 5% sodium sulfate to neutralize them. Further, samples were washed in water for 1 day, followed by a treatment with 10% thioglycolate (catalog number T0632-25G, Sigma-Aldrich) in 7.5% NBF for 2 days. Following treatment, the samples were once again washed with water and dehydrated. Finally, the samples were embedded in paraffin.

### Histology

Formalin-fixed tissues were cut into 5-μm thick sections using a Microm HM 335E microtome and subsequently collected in a 42 °C water bath. The sections were stained with hematoxylin and eosin and afterward photographed with an Olympus BX63 light microscope equipped with a UC-90 camera and the software cellSense Dimension (version: 2.3.18987.0).

### Preparation of riboprobes

Riboprobe templates that anneal to both *X. laevis krt59.L* and *krt59.S* were amplified by RT-PCR from forelimb skin cDNA using the following primer pairs: pan-*krt59* (forward: 5′-GGCGAGAAATCACGTCTGG-3′ and reverse: 5′-CCAGTTTCACATTCATCAGTTCC-3′). The PCR products were purified using the Wizard SV Gel and PCR Clean-Up System (catalog number A9281, Promega) and subsequently ligated into a pGEM-T Easy vector (catalog number A137A, Promega). After propagation in *E. coli* (catalog number L2001, Promega), plasmids containing an insert were isolated using the QIAprep Spin Miniprep Kit 250 (catalog number 27106, Qiagen). Sequencing of the plasmids was performed by Microsynth using SP6 primers to identify the orientation of the inserts. PCR was performed with M13 primers (forward: 5′-CGCCAGGGTTTTCCCAGTCACGAC-3′ and reverse: 5′-CAGGAAACAGCTATGAC-3′) to linearize the plasmids containing the insert in either antisense or sense orientation. For in vitro transcription with the DIG RNA labeling mix (catalog number 11175025910, Roche) the linearized insert containing a T7 RNA polymerase binding site was used as a template. Antisense and sense probes were generated by transcription with T7 RNA polymerase, subsequent precipitation with 0.4 M LiCl (Sigma-Aldrich) and ethanol at −20 °C overnight. Afterward, the probes were washed with 70% and 100% ethanol and resuspended in RNase-free water and finally stored at −80 °C.

### mRNA in situ hybridization

mRNA in situ hybridization was performed on formalin-fixed, paraffin-embedded forelimb skin sections containing nuptial pads from *Xenopus laevis* according to a previously published protocol^48^ with modifications to the blocking step. Sections (5 µm) were prepared using a Microm HM 335E microtome (Thermo Fisher Scientific), floated on a 42 °C water bath, and collected on SuperFrost Plus slides (catalog number J1800AMNZ, Epredia). After incubation at 58 °C for 1 h, sections were deparaffinized in xylene (catalog number 1330-20-7, Fisher Chemical) and rehydrated through a descending ethanol series. Tissue permeability was increased by treatment with proteinase K (20 µg/ml; catalog number 03654672103, Roche) for 10 min at 37 °C. To reduce nonspecific probe binding, sections were incubated sequentially in 1% triethanolamine (catalog number 90279, Sigma-Aldrich) in phosphate-buffered saline (PBS), followed by 1% triethanolamine containing 0.15% and 0.3% acetic anhydride (catalog number 21390293, Prolabo). Samples were then post-fixed in 4% paraformaldehyde (PFA) in PBS for 40 min, dehydrated through an ascending ethanol series, air-dried for 1 h, and prehybridized in hybridization buffer at 60 °C for 2 h. Digoxigenin-labeled sense and antisense riboprobes were used at a concentration of 2 ng/µl. Prior to hybridization, probes were denatured at 85 °C for 10 min in hybridization buffer containing 4 M urea (catalog number 0568, VWR), 5× saline-sodium citrate (SSC; catalog number 10541, Roth), 100 µg/ml heparin (catalog number H3149, Sigma-Aldrich), 5 mM EDTA (catalog number 80401, Roth), 1× Denhardt’s reagent (catalog number D2531, Sigma-Aldrich), 100 µg/ml salmon sperm DNA (catalog number 201190, Agilent), and 5% dextran sulfate (catalog number D8906, Sigma-Aldrich). Hybridization was performed overnight at 60 °C. Excess probe was removed by sequential washes in 4 M urea containing 4×, 2×, and 1× SSC at 58 °C, followed by a final wash in 1× SSC at 37 °C. To block nonspecific antibody binding, sections were incubated for 2 h in 0.1 M maleic acid buffer (MAB, pH 7.5; catalog number M0375, Sigma-Aldrich) supplemented with 2% skim milk (catalog number 70166, Sigma-Aldrich). Hybridized probes were detected using an alkaline phosphatase-conjugated anti-digoxigenin antibody (1:5000; catalog number 11093274910, Roche) diluted in the same blocking solution and incubated overnight at 4 °C. Following washes in alkaline phosphatase buffer (0.5 M Tris-HCl, pH 9.5, 0.5 M NaCl), signal development was carried out in alkaline phosphatase buffer supplemented with 50 mM MgCl₂, 5-bromo-4-chloro-3-indolyl phosphate (BCIP; catalog number 11383221001, Roche) and nitroblue tetrazolium (NBT; catalog number 11383213001, Roche). Stained sections were mounted with Aquatex (catalog number 108562, Sigma-Aldrich) and documented using an Olympus UC-90 digital camera mounted on an Olympus BX63 light microscope and the software cellSense Dimension (version: 2.3.18987.0).

### RNA isolation, RNA-seq, and data analysis

Following the manufacturer’s protocol, RNA was obtained from ventral forelimb skin above the humerus and ventral hindlimb skin above the tibia-fibula of three male wild-type *X. laevis* frogs (5 years old), as well as forelimb toes of two male wild-type and two male Hoxc13 knockout *X. tropicalis* frogs at an age of 7 months using Trizol (catalog number 30-2010, VWR) and a Precellys homogenizer (VWR). Sequencing libraries from total RNA of the *X. laevis* samples were generated at the Core Facility Genomics, Medical University of Vienna, using the NEBNext Poly(A) mRNA Magnetic Isolation Module and the NEBNext UltraExpress RNA Library Prep Kit for Illumina with Unique Dual Indices (UDIs) according to the user manual (New England Biolabs). Libraries were QC-checked on a Bioanalyzer 2100 (Agilent) using a High Sensitivity DNA Kit for correct insert size and quantified using Qubit dsDNA HS Assay (Invitrogen). Sequencing of the libraries was performed on a P2 flowcell of a NextSeq2000 instrument in 1×50 bp single-end sequencing mode. On average, 17 million reads per sample were generated. Reads in fastq format were generated using the Illumina bcl2fastq command line tool (v2.19.1.403) without trimming of the sequencing adapters. Sequencing libraries from the total RNA of the *X. tropicalis* samples were generated by Novogene Co. (Nanjing, China). Messenger RNA (mRNA) was purified from total RNA using poly-T oligo-attached magnetic beads. Fragmented mRNA was reverse transcribed using random hexamer primers to synthesize first-strand cDNA, followed by second-strand synthesis. Libraries were prepared through end repair, A-tailing, adapter ligation, size selection, amplification, and purification. Libraries were quantified using Qubit and real-time PCR, and size distribution was assessed using a Bioanalyzer. Libraries were sequenced on an Illumina platform NovaSeq X Plus Series, generating paired-end 150 bp (PE150) reads. The number of raw reads ranged from 29 million to 100 million per sample.

The RNA-seq data were analyzed according to a published protocol^13^. Briefly, the reads were mapped to the *X. laevis* and *X. tropicalis* reference genomes Xenopus_laevis_v10.1, NCBI (accession number: GCA_017654675.1) and UCB_Xtro_10.0, NCBI (accession number: GCA_000004195.4), respectively. Read counts were calculated using salmon (version: 1.10.3)^49^ and differential gene expression analysis was performed with the Python-implemented module DESeq2 (PyDESeq2)^50^ in Python (version: 3.10). To assess statistical significance, the Wald test, followed by the Benjamini–Hochberg correction for multiple testing, was performed^51^. Differential expression results from *X. laevis* forelimb skin v*ersus* hindlimb skin RNA-seq (Supplementary Data S1) were visualized in a volcano plot (Fig. 2), while keratin gene expression in forelimb toes of wild-type and *hoxc13* knockout *X. tropicalis* was displayed in a clustermap using a cube root-transformed color scale (Fig. 4C). Both visualizations were generated using the Python packages pandas (version: 2.3.2), numpy (version: 2.3.3), seaborn (version: 0.13.2), and matplotlib (version: 3.10.6). Gene Ontology (GO) enrichment analysis was performed based on functional annotation of the *X. laevis* proteome Xenopus_laevis_v10.1 (NCBI accession GCA_017654675.1) to GO terms using eggNOG-mapper (version: 2)^52^, and statistical overrepresentation of GO categories among differentially expressed genes was assessed with GOATOOLS (version: 1.5.2) Python package^53^.

### Proteomics

The freshly dissected tissue sample intended for proteomic analysis was placed in 200 μl of lysis buffer containing 30 mM Tris, 7 M urea (catalog number 0568, VWR), 2 M thiourea (catalog number T7875, Sigma-Aldrich), 4% CHAPSO (catalog number 28304, Pierce), and 0.2 M dithiothreitol (DTT). The sample was incubated at 70 °C for 3 h, homogenized using a Precellys homogenizer (VWR), and then centrifuged at 18,000× *g* for 15 min at 4 °C. The resulting supernatant was collected for mass spectrometry-based proteomics according to a published protocol^13^. The protein reference database for *X. laevis* (taxonomy ID: 8355, reference proteome: UP000186698) was downloaded from UniProt. Peptides were identified with Proteome Discoverer Software (version: 2.4.1.15).

### Statistics and reproducibility

Differential gene expression analysis of forelimb (*n* = 3) *versus* hindlimb (*n* = 3) skin of *X. laevis* and wild-type (*n* = 2) *versus hoxc13* knockout (*n* = 2) *X. tropicalis* forelimb toes was performed using DESeq2 implemented in Python (PyDESeq2)^50^. DESeq2 has been designed for the analysis of high-throughput sequencing data with small numbers of samples. To overcome the limitations associated with the analysis of individual genes, the variance information across all genes is used to obtain stabilized variance estimates for individual genes^54,55^. The Wald test, followed by the Benjamini–Hochberg correction for multiple testing, was performed to calculate *P* values and adjusted *P* values (*P*adj)^51^. Cornified spine densities in male (*n* = 3) and female (*n* = 3) *X. laevis* frogs were analyzed by a two-sided *t* test.

### Reporting summary

Further information on research design is available in the Nature Portfolio Reporting Summary linked to this article.

## Supplementary information

Supplementary Material

Description of Additional Supplementary Files

Supplementary Data S1

Supplementary Data S2

Supplementary Data S3

Reporting Summary

Transparent Peer Review file

## Data Availability

Sequence data that support the findings of this study have been deposited in GenBank under the accession codes PRJNA1335124 and PRJNA1344275. The mass spectrometry-based proteomics data have been deposited in the PRIDE repository with the project accession PXD071004. Publicly available RNA-seq data from *X. tropicalis* claws are available in NCBI under accession code PRJNA1055414^13^. Gene Ontology annotations used for functional enrichment analyses were obtained from the official Gene Ontology website (http://geneontology.org/). The reference genomes used for RNA-seq analyses, *X. tropicalis* UCB_Xtro_10.0 (NCBI accession GCA_000004195.4) and *X. laevis* Xenopus_laevis_v10.1 (NCBI accession GCA_017654675.1), are available through NCBI. The protein reference database for *X. laevis* (taxonomy ID: 8355, reference proteome: UP000186698) was downloaded from UniProt. Source data of Fig. 2 is provided in the Supplementary Data S1 file. The plasmid used for riboprobe generation has been deposited in Addgene with the ID 258303.
