# Supplementary material for: Hoxc13 and homologs of mammalian hair keratins are required for the cornification of nuptial pads in Xenopus frogs
Source: Commun Biol. 2026 Jun 20;9:1302. doi: 10.1038/s42003-026-10519-y (PMC13642941; doi:10.1038/s42003-026-10519-y)
Supplement: Supplementary file 1 [file 42003_2026_10519_MOESM1_ESM.pdf]

Supplementary Material: Supplementary Tables and Figures

Hoxc13 and homologs of mammalian hair keratins are required for the cornification of nuptial pads in Xenopus frogs

Julia Steinbinder, Attila Placido Sachslehner, Bahar Golabi, Marjolein Carron, Kris Vleminckx, Leopold Eckhart

Content

Supplementary Figures S1-S5

Supplementary Tables S1-S7

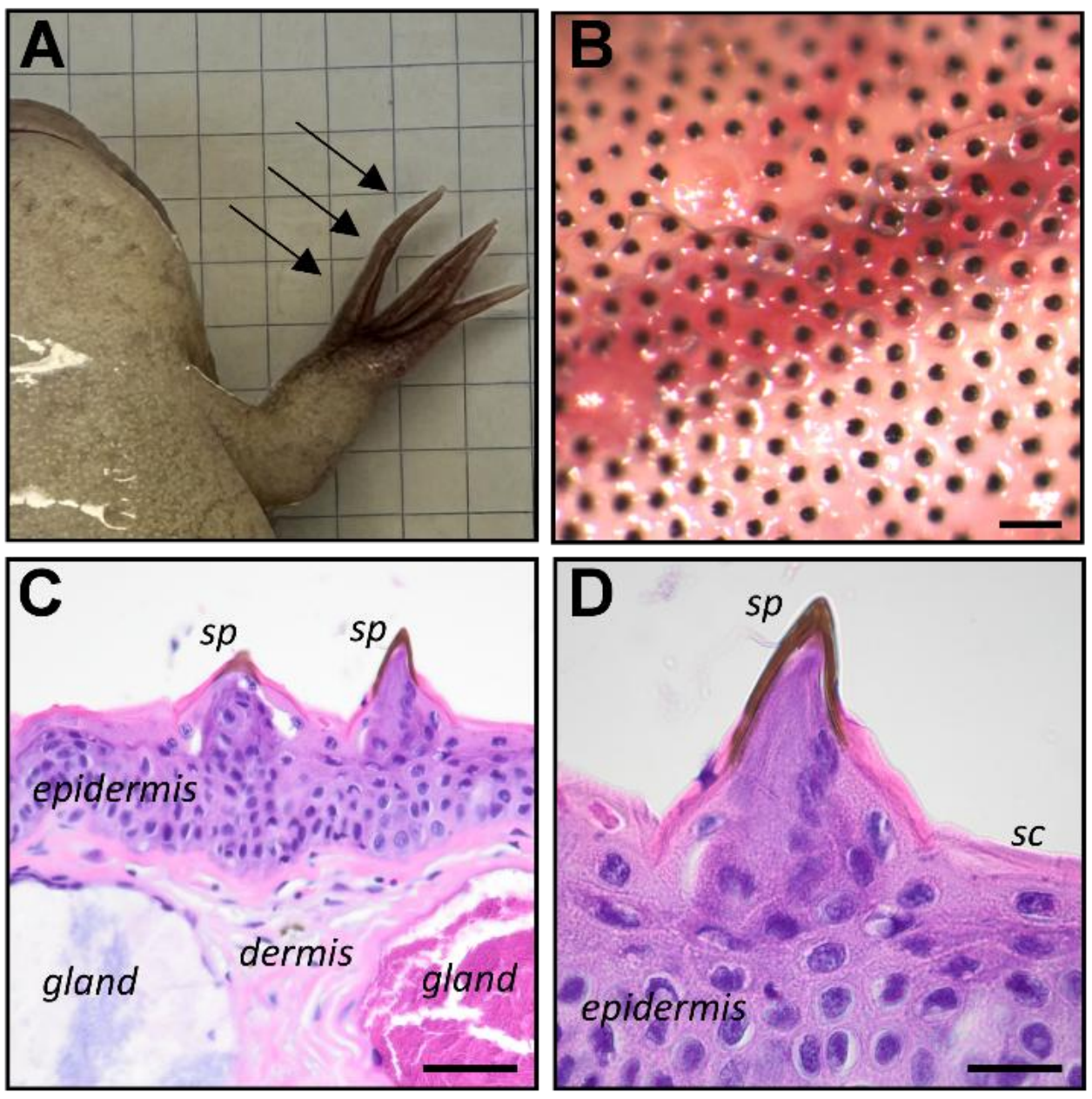

Supplementary Figure S1. Cornified spines on female *Xenopus* frog forelimb toes. **(A)** Macroscopic image of a forelimb of a female *X. laevis* frog at an age of 5 years. Arrows point to the cornified spines. The side length of the squares in the background is 5 mm. **(B)** Stereomicroscopic image of female *X. laevis* cornified spines. **(C, D)** Hematoxylin and eosin (H&E) staining of cornified spines of female *X. laevis* frogs. Spines (sp) are indicated. Scale bars: 100 µm **(B)**, 50 µm **(C)**, 20 µm **(D)**.

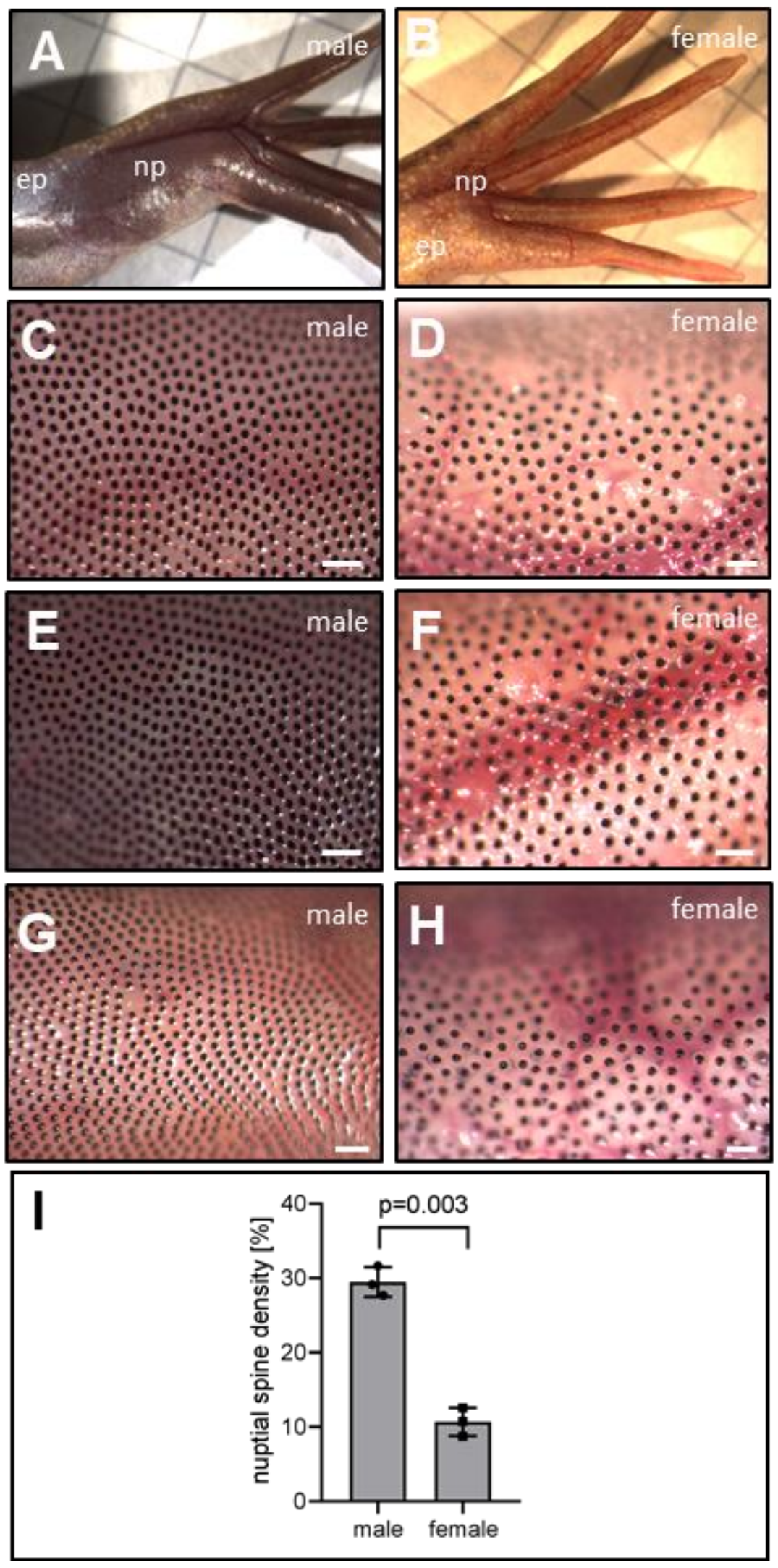

Supplementary Figure S2. Sexual dimorphism of cornified spine density in *X. laevis***.** Macroscopic images of the forelimbs of male **(A)** and female **(B)** *X. laevis* frogs. Stereomicroscopic images of the nuptial pads of three male frogs **(C, E, G)** and nuptial pad-like structures of three female frogs **(D, F, H)** were obtained at the same magnification. Note that parts of panel **F** and **G** are shown in Supplementary Figure S1B and Figure 1B, respectively. **(I)** Quantification of spine density defined as the percentage of the surface area covered by pigmented epidermal spines in males and females. Bars indicate the means and error bars indicate standard deviations. The p-value was calculated by a two-sided t-test with n=3 biologically independent samples. Source data underlying the quantification of spine density are provided in Supplementary Data S3. Scale bars: 100 µm **(C-H)**.

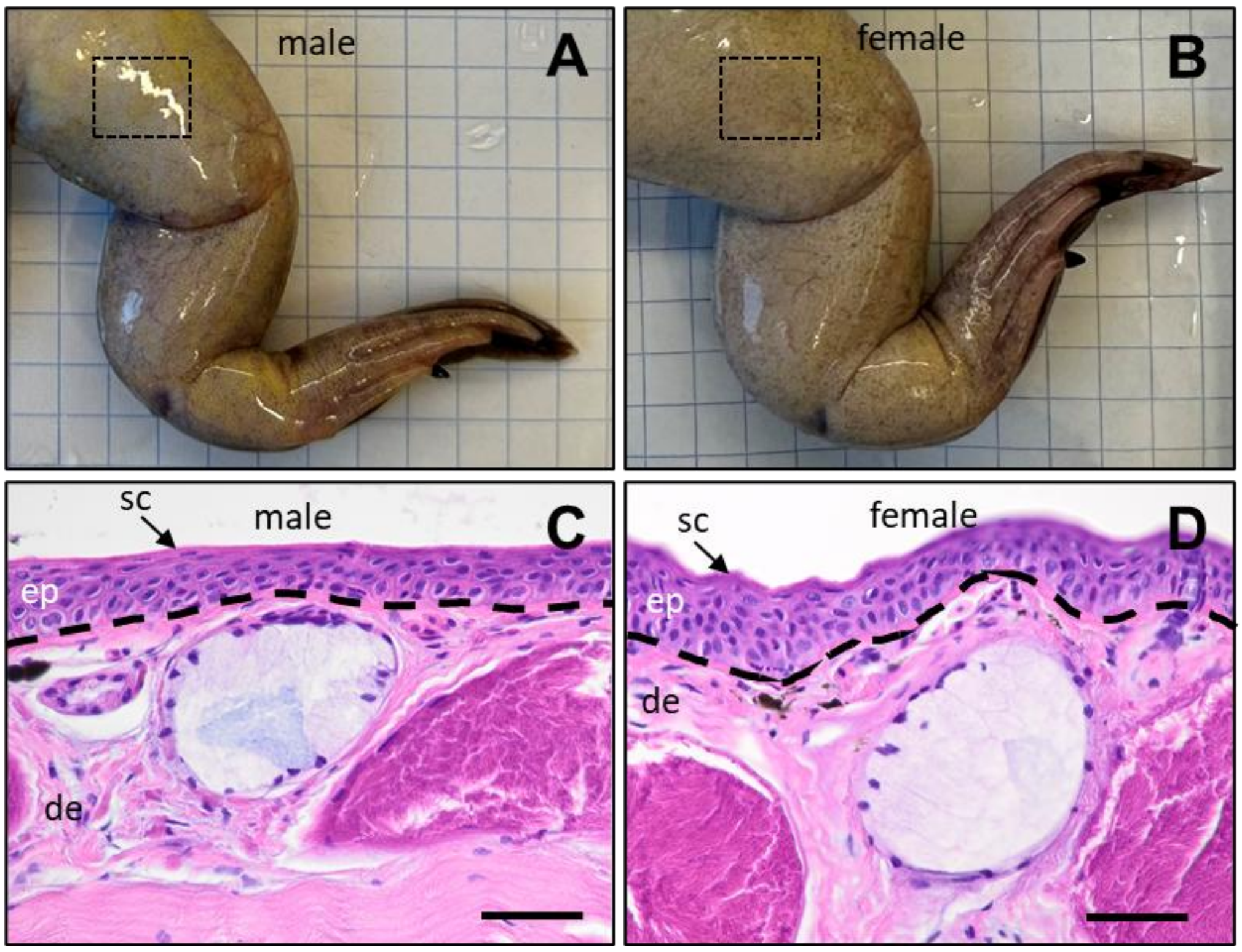

Supplementary Figure S3. Hindlimb skin of male and female *X. laevis* frogs lacks cornified spine structures. Macroscopic images of the ventral hindlimb skin of male **(A)** and female **(B)** frogs. Dashed squares indicate the regions collected for histological and transcriptomic analyses. Side lengths of blue squares in the background **(A, B)**: 5 mm. Hematoxylin and eosin (H&E) staining of hindlimb skin sections from male **(C)** and female **(D)** *X. laevis* frogs showing a stratified epidermis without cornified projections or pigmented spines. The dashed line separates the epidermis (ep) from the underlying dermis (de). Abbreviations: ep, epidermis; de, dermis; sc, stratum corneum. Scale bars **(C, D)**: 50 µm.

A

hoxc13 mutations: nucleotide sequences

```
                                         ▼
guide RNA                               CCAGGCAATGCACGGCTCCC
wildtype                   TCTCCAGAAGCCGCCAGGCAATGCACGGCTCCCCCGTCTTCTTCC
                            S  P  E  A  A  R  Q  C  T  A  P  P  S  S  S

hoxc13 KO1 allele 1        TCTCCAGAAGCCGC-----AATGCACGGCTCCCCCGTCTTCTTCC
                            S  P  E  A  A        M  H  G  S  P  V  F  F

hoxc13 KO1 allele 2        TCTCCAGAAGCCGCC----AATGCACGGCTCCCCCGTCTTCTTCC
                            S  P  E  A  A       N  A  R  L  P  R  L  L  P

hoxc13 KO2 allele 1        TCTCCAGAAGCCGC-----AATGCACGGCTCCCCCGTCTTCTTCC
                            S  P  E  A  A        M  H  G  S  P  V  F  F

hoxc13 KO2 allele 2        TCTCCAGAAGCCGCCAGAAGCAATGCACGGCTCCCCCGTCTTCTT
                            S  P  E  A  A  R  S  N  A  R  L  P  R  L  L
```

B

Impact of mutations on the Hoxc13 protein structure

```
                                                    <                                         HoxA13_N domain
wildtype              MTTSLILHPHWADTLMYVYETSPNEKNPRKSPAMEGLSGNCASSHCRDFISHPALGRHSSAIGSHQGPVYTDITSPEAAR-QCTAPPSSSNASLGYAYPFGSS
Hoxc13 KO1 allele 1   MTTSLILHPHWADTLMYVYETSPNEKNPRKSPAMEGLSGNCASSHCRDFISHPALGRHSSAIGSHQGPVYTDITSPEAAM-HGSPVFFQRLPGLCLPIWKQLL
Hoxc13 KO1 allele 2   MTTSLILHPHWADTLMYVYETSPNEKNPRKSPAMEGLSGNCASSHCRDFISHPALGRHSSAIGSHQGPVYTDITSPEAA--NARLPRLLPTPPWAMPTHLEAA
Hoxc13 KO2 allele 1   MTTSLILHPHWADTLMYVYETSPNEKNPRKSPAMEGLSGNCASSHCRDFISHPALGRHSSAIGSHQGPVYTDITSPEAAM-HGSPVFFQRLPGLCLPIWKQLL
Hoxc13 KO2 allele 2   MTTSLILHPHWADTLMYVYETSPNEKNPRKSPAMEGLSGNCASSHCRDFISHPALGRHSSAIGSHQGPVYTDITSPEAARSNARLPRLLPTPPWAMPTHLEAA

                                     HoxA13_N domain          >
wildtype              YYGCRLSQSHNVNLHQKPCSYHPAEKYPEPSNPLPSEEFSSRAKEFAFYPSFASSYQAVPGYLDMSVVPGISSHPEPRHDTLLSMEGYQHWALPNGWDGQVYC
Hoxc13 KO1 allele 1   RLQALSVPQRQLTPETLLLPSGREIPRAQQPLAQRRILLQGQGICFLP*------------------------------------------------------
Hoxc13 KO1 allele 2   ITAAGSLSPTTSTYTRNPAPTIRPRNTPSPATPCPAKNSPPGPRNLLFTLVLPAPTRRSLVTWTCQ*------------------------------------
Hoxc13 KO2 allele 1   RLQALSVPQRQLTPETLLLPSGREIPRAQQPLAQRRILLQGQGICFLP*------------------------------------------------------
Hoxc13 KO2 allele 2   ITAAGSLSPTTSTYTRNPAPTIRPRNTPSPATPCPAKNSPPGPRNLLFTLVLPAPTRRSLVTWTCQ*------------------------------------

                                                    <                Homeobox domain                    >
wildtype              SKDQSQSGHLWKAPFPDVVPLQPEVSNYRRGRKKRVPYTKIQLKELEKEYAASKFITKEKRRRISTATSLSERQVTIWFQNRRVKEKKVVTKCKTAHLHNT
Hoxc13 KO1 allele 1   -----------------------------------------------------------------------------------------------------
Hoxc13 KO1 allele 2   -----------------------------------------------------------------------------------------------------
Hoxc13 KO2 allele 1   -----------------------------------------------------------------------------------------------------
Hoxc13 KO2 allele 2   -----------------------------------------------------------------------------------------------------
```

Supplementary Figure S4. Characterization of *hoxc13* mutant alleles and their predicted effects on Hoxc13 protein structure. **(A)** Nucleotide sequences of the wildtype *hoxc13* area spanning the guide RNA target site together with the corresponding mutant alleles identified in the two *X. tropicalis* Hoxc13 knockout frogs, KO1 and KO2. Note that hindlimb toes of the same mutant frogs were investigated in a previous study (Carron et al. 2024). The guide RNA sequence (Chr2:146200810-146200829 in XenTro10.0) and the surrounding sequences are shown in reverse-complement together with the encoded amino acid sequence. Deleted nucleotides are indicated by red dashes, whereas inserted bases are displayed in red text. Predicted amino acid translations for each allele are shown beneath the nucleotide sequences. Underlined letters denote the protospacer adjacent motif (PAM). The arrowhead indicates the predicted cut site. **(B)** Effect of the mutations on Hoxc13 on the protein level. Amino acid sequences of wildtype Hoxc13 were aligned with the sequences encoded by alleles 1 and 2 of knockout frogs KO1 and KO2. Residues translated after frameshift mutations are shown in red. Premature stop codons are marked by asterisks. The HoxA13_N domain is indicated by a single underline, whereas the homeobox domain is marked by a double underline. This figure was modified from supplementary figure 2 of Carron et al. (2024).

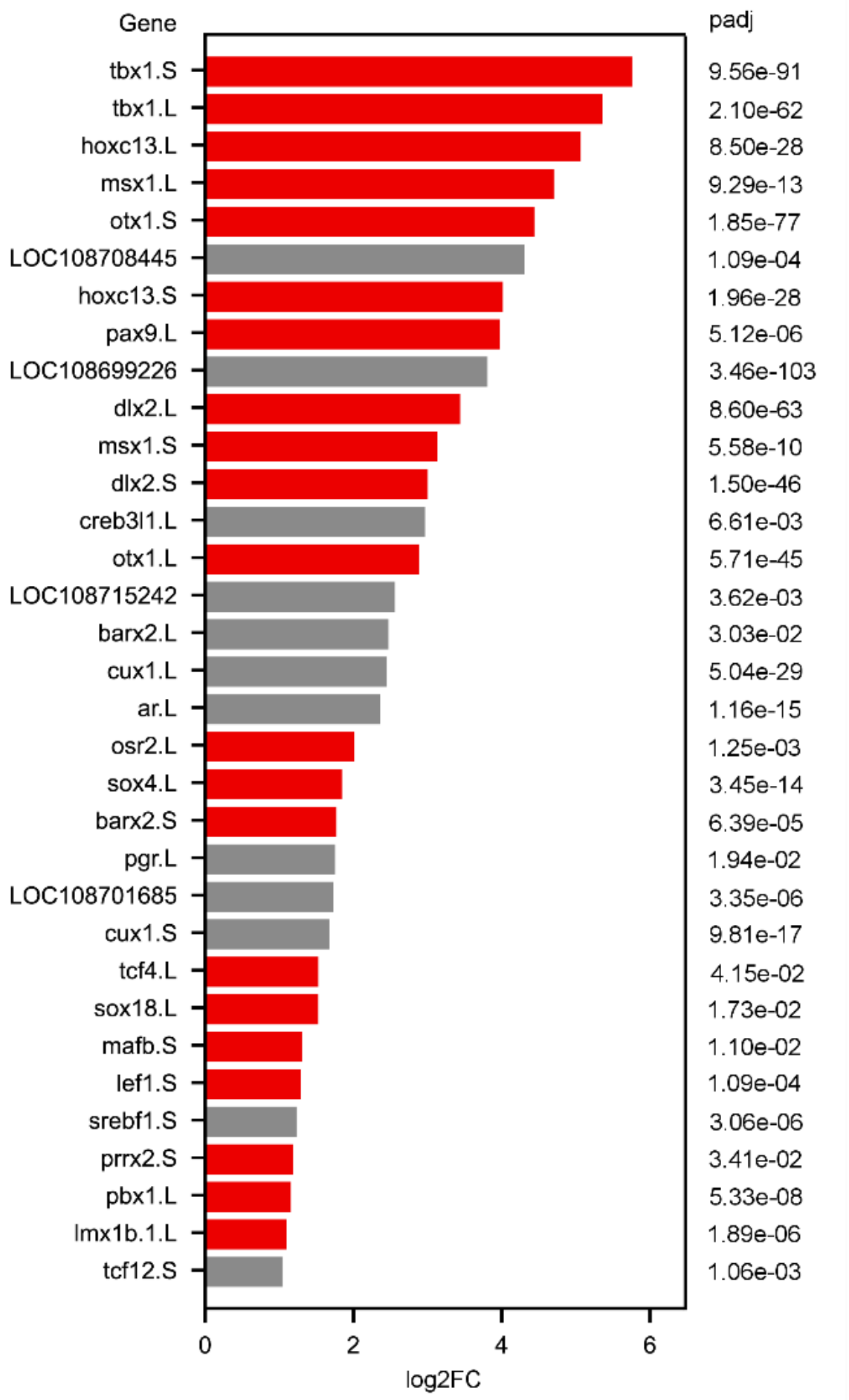

Supplementary Figure S5. Transcription factors upregulated in skin with nuptial pads relative to skin without nuptial pads. The bar chart shows transcription factors belonging to the significantly enriched Gene Ontology (GO) category "DNA-binding transcription activator activity" (GO:0001216; q<0.05). All genes depicted are significantly upregulated (adjusted P-value, padj<0.05; fold change >2) in forelimb skin containing nuptial pads compared to hindlimb skin lacking these structures with n=3 biologically independent samples per tissue type. Red bars highlight transcription factors known to control placode formation and the development of ectodermal appendages (Suppl. Table S6).

Supplementary Table S1. Expression levels (normalized counts, RNA-seq) of keratin genes in wildtype (wt) and Hoxc13-deficient (KO) *X. tropicalis* forelimb toes, corresponding to Figure 4C.

Gene	forelimb toe wt1	forelimb toe wt2	forelimb toe *hoxc13* KO1	forelimb toe *hoxc13* KO2	log2FC	lfcSE	stat	padj
krt59	20209	75672	3	7	13.17	0.70	18.75	1.03E-74
krt9.1	84325	134427	1088	5	7.64	2.27	3.37	3.13E-02
krt24	22443	138032	41618	9290	1.66	1.41	1.17	1.00E+00
krt17	33697	82708	19186	8107	2.09	0.51	4.14	2.77E-03
krt61	61492	163240	10186	6715	3.73	0.47	7.89	1.49E-12
krt78.6	233553	379178	40085	27179	3.19	0.40	7.93	1.18E-12
krt50	254199	403261	11028	3768	5.47	0.49	11.16	8.96E-26
krt78.2	140461	112136	194172	178684	-0.56	0.36	-1.58	7.98E-01
krt57	204282	108827	203004	169179	-0.25	0.41	-0.61	1.00E+00
krt12.6	119651	89440	153897	123647	-0.41	0.37	-1.11	1.00E+00
krt78.9	109125	202405	301990	244638	-0.81	0.41	-2.00	5.09E-01
krt55	51789	31055	62081	69945	-0.67	0.39	-1.73	6.98E-01
krt78.7	967462	888358	727372	1039990	0.07	0.37	0.19	1.00E+00
krt53	878248	902923	1264275	1408801	-0.59	0.35	-1.69	7.36E-01

*Notes: wt, wildtype; *hoxc13* KO, *hoxc13* knockout; log2FC, log2 fold change; lfcSE, standard error of the estimated log2 fold change; stat, Wald test statistic (Z-value) with n=2 biologically independent samples per condition was computed as the ratio of the estimated log2 fold change to its standard error; Padj, adjusted p-value; n.a., not applicable.

Supplementary Table S2. Expression levels (normalized counts, RNA-seq) of keratin genes in wild-type (wt) and Hoxc13-deficient (KO) *X. tropicalis* forelimb toes.

Gene	forelimb toe wt1	forelimb toe wt2	forelimb toe hoxc13 KO1	forelimb toe hoxc13 KO2	log2FC	lfcSE	stat	padj
krt59	20209	75672	3	7	13.17	0.70	18.75	1.03E-74
krt9.1	84325	134427	1088	5	7.64	2.27	3.37	3.13E-02
krt56	0	48	0	0	7.00	3.05	2.29	3.22E-01
krt50	254199	403261	11028	3768	5.47	0.49	11.16	8.96E-26
krt61	61492	163240	10186	6715	3.73	0.47	7.89	1.49E-12
krt12.1	0	4	0	0	3.22	5.20	0.62	n.a.
krt78.6	233553	379178	40085	27179	3.19	0.40	7.93	1.18E-12
krt15.1	0	2	0	0	2.50	5.24	0.48	n.a.
krt17	33697	82708	19186	8107	2.09	0.51	4.14	2.77E-03
krt78.3	1105	27150	6886	318	1.97	2.19	0.90	1.00E+00
krt20	1	0	0	0	1.68	5.31	0.32	n.a.
krt24	22443	138032	41618	9290	1.66	1.41	1.17	1.00E+00
krt23	702	1388	262	587	1.30	0.50	2.58	1.92E-01
krt80	2154	2466	1199	1550	0.75	0.38	1.99	5.09E-01
krt62	30	85	60	29	0.39	0.84	0.47	1.00E+00
krt78.4	5979	23046	13200	10319	0.30	0.52	0.58	1.00E+00
krt15.2	1	0	1	0	0.25	4.96	0.05	n.a.
krt18.1	479	550	478	408	0.22	0.41	0.52	1.00E+00
krt78.1	24	48	52	16	0.10	0.96	0.11	1.00E+00
krt222	5	16	18	2	0.09	1.61	0.06	1.00E+00
krt78.7	967462	888358	727372	1039990	0.07	0.37	0.19	1.00E+00
krt34	13	23	11	24	0.04	1.18	0.03	1.00E+00
krt9.2	582	521	685	510	-0.12	0.41	-0.28	1.00E+00
krt12.2	7	4	7	5	-0.21	1.90	-0.11	n.a.
krt57	204282	108827	203004	169179	-0.25	0.41	-0.61	1.00E+00
krt7	1314	2621	2977	2105	-0.37	0.44	-0.84	1.00E+00
krt8.1	1343	1573	2467	1328	-0.38	0.42	-0.90	1.00E+00
krt12.6	119651	89440	153897	123647	-0.41	0.37	-1.11	1.00E+00
krt18.3	208	132	239	217	-0.43	0.50	-0.85	1.00E+00
krt19	2702	2687	4847	2527	-0.45	0.41	-1.09	1.00E+00
krt78.2	140461	112136	194172	178684	-0.56	0.36	-1.58	7.98E-01
krt53	878248	902923	1264275	1408801	-0.59	0.35	-1.69	7.36E-01
krt55	51789	31055	62081	69945	-0.67	0.39	-1.73	6.98E-01
krt78.9	109125	202405	301990	244638	-0.81	0.41	-2.00	5.09E-01
krt78.5	104	139	193	282	-0.96	0.52	-1.86	6.02E-01
krt6b	437	121	265	953	-1.13	0.65	-1.75	6.87E-01
krt8.2	129	153	243	406	-1.20	0.50	-2.41	2.67E-01
krt12.5	62	87	188	226	-1.47	0.54	-2.70	1.53E-01
krt18.2	12	0	35	2	-1.71	1.84	-0.93	1.00E+00
krt54	985	569	2801	3753	-2.08	0.42	-5.00	7.74E-05
krt12.3	0	1	3	4	-3.03	3.52	-0.86	n.a.
krt70	0	1	9	4	-3.12	2.68	-1.16	n.a.

*Notes: wt, wildtype; *hoxc13* KO, *hoxc13* knockout; log2FC, log2 fold change; lfcSE, standard error of the estimated log2 fold change; stat, Wald test statistic (Z-value) with n=2 biologically independent samples per condition was computed as the ratio of the estimated log2 fold change to its standard error; Padj, adjusted p-value; n.a., not applicable.

Supplement Table S3. Expression levels (normalized counts, RNA-seq) of *hox* genes in wild-type (wt) and Hoxc13-deficient (KO) *X. tropicalis* forelimb toes.

Gene	forelimb toe wt1	forelimb toe wt2	forelimb toe *hoxc13* KO1	forelimb toe *hoxc13* KO2	log2FC	lfcSE	stat	padj
hoxa1	3	0	17	4	-3.16	2.25	-1.40	n.a.
hoxa11	51	54	94	47	-0.42	0.72	-0.58	1.00E+00
hoxa13	251	327	447	226	-0.22	0.50	-0.44	1.00E+00
hoxa2	38	23	103	43	-1.26	0.81	-1.56	8.16E-01
hoxa3	219	139	309	164	-0.41	0.54	-0.76	1.00E+00
hoxa4	128	69	132	43	0.16	0.71	0.23	1.00E+00
hoxa5	11	6	31	15	-1.54	1.27	-1.21	1.00E+00
hoxa6	5	4	1	3	1.06	2.52	0.42	n.a.
hoxa7	20	20	48	23	-0.87	0.98	-0.89	1.00E+00
hoxa9	185	141	234	141	-0.21	0.53	-0.39	1.00E+00
hoxb2	11	13	31	12	-0.85	1.23	-0.69	1.00E+00
hoxb3	69	57	90	88	-0.50	0.64	-0.79	1.00E+00
hoxb4	0	1	2	4	-2.07	3.46	-0.60	n.a.
hoxb5	0	2	0	1	1.03	4.88	0.21	n.a.
hoxb6	99	85	101	100	-0.13	0.59	-0.23	1.00E+00
hoxb7	38	29	45	32	-0.20	0.86	-0.23	1.00E+00
hoxb8	18	15	19	12	0.13	1.21	0.11	1.00E+00
hoxb9	4	10	0	15	-0.10	2.07	-0.05	n.a.
hoxc10	3	10	19	0	-0.61	2.03	-0.30	n.a.
hoxc11	0	0	95	2	-8.10	2.73	-2.97	8.80E-02
hoxc12	1	5	49	22	-3.45	1.28	-2.70	1.52E-01
hoxc13	1141	4083	1220	592	1.53	0.55	2.76	1.37E-01
hoxc4	24	6	25	10	-0.24	1.29	-0.18	1.00E+00
hoxc5	0	1	2	0	-1.19	4.92	-0.24	n.a.
hoxc6	0	0	1	0	-1.54	5.35	-0.29	n.a.
hoxc8	1	4	81	13	-4.20	1.30	-3.22	4.78E-02
hoxc9	0	0	21	1	-5.98	3.29	-1.82	n.a.
hoxd10	161	275	139	280	0.06	0.55	0.11	1.00E+00
hoxd13	320	612	496	500	-0.09	0.46	-0.20	1.00E+00
hoxd3	67	102	82	79	0.08	0.64	0.13	1.00E+00
hoxd4	12	12	8	15	0.05	1.40	0.04	1.00E+00
hoxd8	32	28	34	43	-0.37	0.87	-0.42	1.00E+00
hoxd9	76	84	120	83	-0.34	0.61	-0.55	1.00E+00

*Note: Mutation of *hoxc13* does not suppress the expression of *hoxc13* at the mRNA level but abolishes the Hoxc13 protein in Hoxc13-deficient frogs; log2FC, log2 fold change; lfcSE, standard error of the estimated log2 fold change; stat, Wald test statistic (Z-value) with n=2 biologically independent samples per condition was computed as the ratio of the estimated log2 fold change to its standard error; Padj, adjusted p-value; n.a., not applicable.

Supplement Table S4. Expression levels of *hox* genes in hindlimb toes I-III of wildtype (wt) and *hoxc13*-mutant (KO) *X. tropicalis* frogs

Gene	Hindlimb toes I-III (normalized counts, RNA-seq)			Hindlimb toes I-III (normalized counts, RNA-seq)			log2FC	lfcSE	stat	Padj
	wt1	wt2	wt3	*hoxc13* KO1	*hoxc13* KO2	*hoxc13* KO3				
hoxa1	3	2	8	11	0	6	0.48	1.5	0.3	n.a.
hoxa11	32	70	44	34	63	50	0.01	0.5	0.0	9.99E-01
hoxa13	177	213	112	249	280	185	0.51	0.4	1.5	6.83E-01
hoxa2	15	15	11	8	46	11	0.66	0.8	0.8	8.73E-01
hoxa3	196	201	203	288	300	259	0.50	0.2	2.3	3.26E-01
hoxa4	49	12	28	11	17	44	-0.30	0.8	-0.4	9.55E-01
hoxa5	153	52	114	97	122	192	0.36	0.5	0.7	8.87E-01
hoxa6	36	5	32	4	24	34	-0.26	1.0	-0.3	9.73E-01
hoxa7	125	84	135	91	131	93	-0.12	0.4	-0.4	9.60E-01
hoxa9	590	645	710	632	703	712	0.07	0.2	0.4	9.57E-01
hoxb1	0	0	0	0	0	2	2.22	4.3	0.5	n.a.
hoxb2	4	4	3	11	3	1	0.48	1.2	0.4	n.a.
hoxb3	23	34	9	22	25	12	-0.13	0.7	-0.2	9.81E-01
hoxb4	1	0	0	0	0	2	1.26	4.1	0.3	n.a.
hoxb5	2	0	2	3	1	0	0.08	2.3	0.0	n.a.
hoxb6	49	61	36	56	42	41	-0.07	0.4	-0.2	9.84E-01
hoxb7	6	14	11	17	35	21	1.22	0.7	1.8	5.50E-01
hoxb8	5	4	3	2	17	2	0.85	1.3	0.7	n.a.
hoxb9	14	21	9	10	12	18	-0.14	0.7	-0.2	9.81E-01
hoxc10	4	5	1	21	16	5	2.04	1.0	2.0	4.81E-01
hoxc11	0	0	6	122	561	22	6.95	2.1	3.3	5.92E-02
hoxc12	1	1	17	106	318	46	4.63	1.1	4.4	2.32E-03
hoxc13	910	3999	1308	1319	3403	880	-0.15	0.6	-0.2	9.77E-01
hoxc3	0	0	0	0	0	1	1.15	4.4	0.3	n.a.
hoxc4	23	18	84	58	64	140	1.06	0.7	1.5	6.55E-01
hoxc5	0	0	2	4	1	1	1.86	2.3	0.8	n.a.
hoxc6	0	0	3	3	3	16	2.93	1.8	1.6	n.a.
hoxc8	2	0	104	41	54	129	1.09	2.0	0.5	9.30E-01
hoxc9	0	0	15	36	103	32	3.49	1.4	2.6	2.36E-01
hoxd10	1	0	29	0	0	44	0.57	3.7	0.2	9.85E-01
hoxd13	12	2	108	19	8	134	0.39	1.3	0.3	9.66E-01
hoxd3	22	22	34	30	9	13	-0.59	0.7	-0.9	8.52E-01
hoxd4	2	0	3	0	1	4	0.01	2.3	0.0	n.a.
hoxd8	11	7	11	7	16	5	-0.06	0.8	-0.1	9.90E-01
hoxd9	23	20	13	16	25	29	0.29	0.6	0.5	9.38E-01

*Notes: wt, wildtype; *hoxc13* KO, *hoxc13* knockout; log2FC, log2 fold change; lfcSE, standard error of the estimated log2 fold change; stat, Wald test statistic (Z-value) with n=3 biologically independent samples per tissue type was computed as the ratio of the estimated log2 fold change to its standard error; Padj, adjusted p-value; n.a., not applicable.

Supplementary Table S5. Expression levels (normalized counts, RNA-seq) of appendage-related transcription factors in forelimb toes of wild-type (wt) and Hoxc13-deficient (KO) X. tropicalis frogs.

Gene	forelimb toe wt1	forelimb toe wt2	forelimb toe *hoxc13* KO1	forelimb toe *hoxc13* KO2	log2FC	lfcSE	stat	padj
tbx1	1498	7475	710	360	3.07	0.59	5.16	3.77E-05
dlx2	2155	5511	1142	864	1.93	0.47	4.12	2.96E-03
pax9	95	235	142	0	1.21	3.05	0.40	1.00E+00
otx1	1863	3948	1212	1442	1.13	0.44	2.59	1.89E-01
osr2	426	962	636	285	0.59	0.53	1.12	1.00E+00
msx1	473	909	574	547	0.30	0.45	0.68	1.00E+00
lef1	335	700	452	430	0.23	0.47	0.49	1.00E+00
tcf4	253	363	292	234	0.23	0.47	0.49	1.00E+00
sox18	162	350	608	294	-0.81	0.54	-1.51	8.47E-01

*Notes: wt, wildtype; *hoxc13* KO, *hoxc13* knockout; log2FC, log2 fold change; lfcSE, standard error of the estimated log2 fold change; stat, Wald test statistic (Z-value) with n=2 biologically independent samples per condition was computed as the ratio of the estimated log2 fold change to its standard error; Padj, adjusted p-value; n.a., not applicable.

Supplementary Table S6. Genes annotated with GO:0001216 ("DNA-binding transcription activator activity") enriched in *X. laevis* nuptial pads versus control skin, corresponding to Figure S5.

Gene	log2FC	padj	Notes	References
tbx1.S	5.76	9.56E-91	BMP signalling regulator, expressed in teeth and hair follicle; epidermis-specific Tbx1 knockout altered the hair cycle	[1, 2]
tbx1.L	5.36	2.10E-62	BMP signalling regulator, expressed in teeth and hair follicle; epidermis-specific Tbx1 knockout altered the hair cycle	[1, 2]
hoxc13.L	5.07	8.50E-28	Hoxc13 regulates hair growth	[3]
msx1.L	4.71	9.29E-13	Msx1-deficient mice show delayed hair placode development and abnormal vibrissa follicles	[4]
otx1.S	4.44	1.85E-77	*Otx1* is expressed in vibrissa follicles	[5]
LOC108708445	4.30	1.09E-04	shox.L	
hoxc13.S	4.02	1.96E-28	Hoxc13 regulates hair growth	[3]
pax9.L	3.98	5.12E-06	*pax9* knockout causes loss of keratins in filifom papillae	[6]
LOC108699226	3.81	3.46E-103	MEIS2 is differentially expressed in three layers of the inner root sheath	[7]
dlx2.L	3.44	8.60E-63	DLX2 is expressed in the embryonic epidermis of Xenopus frogs and in avian feather buds alongside Dlx3; Dlx1/2 double mutants have defects in craniofacial ectodermal appendages	[8, 9]
msx1.S	3.13	5.58E-10	Msx1-deficient mice show delayed hair placode development and abnormal vibrissa follicles	[4]
dlx2.S	3.00	1.50E-46	DLX2 is expressed in the embryonic epidermis of Xenopus frogs and in avian feather buds alongside Dlx3; Dlx1/2 double mutants have defects in craniofacial ectodermal appendages	[8, 9]
creb3l1.L	2.97	6.61E-03		
otx1.L	2.88	5.71E-45	*in situ* study showing *Otx1* transcripts in nasal/facial integument, including hair vibrissa follicles	[5]
LOC108715242	2.56	3.62E-03	creb3l1.S	
barx2.L	2.47	3.03E-02	Barx2 deficiency causes hair cycle defects	[10]
cux1.L	2.45	5.04E-29	Cux1 deficiency leads to altered hair follicle morphology	[11, 12]
ar.L	2.36	1.16E-15	androgen receptor	
osr2.L	2.01	1.25E-03	Osr2 controls tooth organogenesis; it interacts with both Msx1 and Pax9 to pattern the tooth developmental field	[13, 14]
sox4.L	1.84	3.45E-14	Mutation in SOX4 is associated with syndromic tooth agenesis	[15]
barx2.S	1.77	6.39E-05	Barx2 deficiency causes hair cycle defects	[10]
pgr.L	1.75	1.94E-02	progesterone receptor	
LOC108701685	1.73	3.35E-06	srebf1	
cux1.S	1.68	9.81E-17	Cux1 deficiency leads to altered hair follicle morphology	[11, 12]
tcf4.L	1.53	4.15E-02	regulates proliferation and secretory activity of dermal papilla cells; coactivator of cux1	[16]
sox18.L	1.52	1.73E-02	Mutations in *Sox18* causes hair follicle defects	[17]
mafb.S	1.30	1.10E-02	MafB regulates hair follicle stem cell activation and hair regeneration; expressed in the inner root sheath	[18, 19]
lef1.S	1.29	1.09E-04	LEF1 controls development of ectodermal appendages such as teeth, vibrissae, hair, and mammary glands	[20, 21]
srebf1.S	1.23	3.06E-06	sterol regulatory element binding transcription factor 1	

prrx2.S	1.18	3.41E-02	potential regulatory factors DC (dermal condensate) and DP (dermal papillae)	[22]
pbx1.L	1.16	5.33E-08	PBX1 attenuates hair follicle-derived mesenchymal stem cell senescence and apoptosis	[23]
lmx1b.1.L	1.10	1.89E-06	causes Nail-Patella Syndrome	[24]
tcf12.S	1.04	1.06E-03	transcription factor 12	

*Notes: log2FC, log2 fold change; padj, adjusted p-value.

Supplementary Table S7. Expression levels (normalized counts, RNA-seq) of selected transcription factors detected in nuptial pads and claws of *X. tropicalis*.

Gene	Nuptial pad 1	Nuptial pad 2	Claw 1	Claw 2	log2FC np:claw
pax9	77	191	4	0	6.19
tbx1	1219	6076	141	55	5.21
meis2	1201	4711	218	38	4.53
shox	168	317	12	12	4.35
dlx2	1753	4479	206	328	3.55
lmx1b.1	652	1672	146	131	3.07
otx1	1516	3209	311	318	2.91
sox4	750	1796	258	185	2.53
msx1	385	739	101	121	2.34
osr2	347	782	221	59	2.01
ar	533	557	154	131	1.94
barx2	2010	6147	1381	1135	1.70
tfap2e	178	183	88	35	1.55
pbx1	855	1476	460	452	1.36
mafb	2622	4962	1286	2471	1.01
tcf12	815	908	488	437	0.90
sox18	132	285	82	147	0.87
tcf4	206	295	140	139	0.85
prrx2	992	1248	574	717	0.80
lef1	273	569	222	328	0.62
maf	105	55	95	28	0.37
nr4a1	1543	682	802	977	0.32
sox21	428	350	488	143	0.30
myocd	56	140	73	98	0.20
srebf1	732	1075	795	925	0.07
cux1	4151	9202	3478	9636	0.03
creb3l1	71	266	149	185	0.02
pou2f3	1344	537	1299	679	-0.07
irf1	2518	807	3022	958	-0.26
pgr	38	15	32	50	-0.65
hoxc13	928	3319	1230	5545	-0.67
creb5	58	68	55	156	-0.74
cebpd	5471	1858	7240	5413	-0.79
gata3	103	113	219	181	-0.88
hoxa1	2	0	4	3	-1.60
esrrg	30	3	85	23	-1.74
foxi1	174	52	897	441	-2.58
hoxa9	150	114	797	895	-2.68
hoxa7	16	16	169	116	-3.16
hoxa5	9	5	207	71	-4.45

*Notes: The transcription factors were selected as homologs of the genes linked to the GO term “DNA-binding transcription activator activity” in *X. laevis* nuptial pads. Nuptial pad, forelimb toe with nuptial pads; Claw, hindlimb inner toe with claw. np, nuptial pad. Claw expression data is available on NCBI under accession code PRJNA1055414.

Supplementary references

1. Chen, T. *et al.* An RNA interference screen uncovers a new molecule in stem cell self-renewal and long-term regeneration. *Nature* **485**, 104–108 (2012).
2. Zoupa, M., Seppala, M., Mitsiadis, T. & Cobourne, M. T. Tbx1 is expressed at multiple sites of epithelial-mesenchymal interaction during early development of the facial complex. *Int J Dev Biol* **50**, 504–510 (2006).
3. Godwin, A. R. & Capecchi, M. R. Hoxc13 mutant mice lack external hair. *Genes Dev* **12**, 11–20 (1998).
4. Duverger, O. & Morasso, M. I. Role of homeobox genes in the patterning, specification, and differentiation of ectodermal appendages in mammals. *J Cell Physiol* **216**, 337–346 (2008).
5. Kanzler, B. *et al.* Differential expression of two different homeobox gene families during mouse tegument morphogenesis. *Int J Dev Biol* **38**, 633–640 (1994).
6. Jonker, L., Kist, R., Aw, A., Wappler, I. & Peters, H. Pax9 is required for filiform papilla development and suppresses skin-specific differentiation of the mammalian tongue epithelium. *Mech Dev* **121**, 1313–1322 (2004).
7. Jave-Suarez, L. F., Winter, H., Langbein, L., Rogers, M. A. & Schweizer, J. HOXC13 is involved in the regulation of human hair keratin gene expression. *J. Biol. Chem.* **277**, 3718–3726 (2002).
8. Morasso, M. I., Jamrich, M. & Sargent, T. D. The homeodomain gene Xenopus Distal-less-like-2 (Xdll-2) is regulated by a conserved mechanism in amphibian and mammalian epidermis. *Dev Biol* **162**, 267–276 (1994).
9. Qiu, M. *et al.* Role of the Dlx homeobox genes in proximodistal patterning of the branchial arches: mutations of Dlx-1, Dlx-2, and Dlx-1 and -2 alter morphogenesis of proximal skeletal and soft tissue structures derived from the first and second arches. *Dev Biol* **185**, 165–184 (1997).
10. Olson, L. E., Zhang, J., Taylor, H., Rose, D. W. & Rosenfeld, M. G. Barx2 functions through distinct corepressor classes to regulate hair follicle remodeling. *PNAS* **102**, 3708–3713 (2005).
11. Tufarelli, C., Fujiwara, Y., Zappulla, D. C. & Neufeld, E. J. Hair Defects and Pup Loss in Mice with Targeted Deletion of the First Cut Repeat Domain of the*Cux/CDP*Homeoprotein Gene. *Dev. Biol* **200**, 69–81 (1998).
12. Luong, M. X. *et al.* Genetic ablation of the CDP/Cux protein C terminus results in hair cycle defects and reduced male fertility. *Mol Cell Biol* **22**, 1424–1437 (2002).
13. Jia, S. *et al.* Bmp4-Msx1 signaling and Osr2 control tooth organogenesis through antagonistic regulation of secreted Wnt antagonists. *Dev Biol* **420**, 110–119 (2016).
14. Zhou, J. *et al.* Osr2 acts downstream of Pax9 and interacts with both Msx1 and Pax9 to pattern the tooth developmental field. *Dev. Biol* **353**, 344–353 (2011).
15. Zhou, M. *et al.* Identification of a novel de novo mutation in SOX4 for syndromic tooth agenesis. *Clin Oral Investig* **28**, 287 (2024).

16. Xiong, Y., Liu, Y., Song, Z., Hao, F. & Yang, X. Identification of Wnt/β-catenin signaling pathway in dermal papilla cells of human scalp hair follicles: TCF4 regulates the proliferation and secretory activity of dermal papilla cell. *J Dermatol* **41**, 84–91 (2014).

17. Pennisi, D. *et al.* Mutations in Sox18 underlie cardiovascular and hair follicle defects in ragged mice. *Nat Genet* **24**, 434–437 (2000).

18. Gao, Q., Zeng, S., Chen, Y., Bao, Y. & Cui, H. MafB regulates hair follicle stem cell activation and hair regeneration through NFATc1 signaling pathway. *Biochem Biophys Res Commun* **772**, 152035 (2025).

19. Miyai, M. *et al.* Transcription Factor MafB Coordinates Epidermal Keratinocyte Differentiation. *J Invest Dermatol* **136**, 1848–1857 (2016).

20. Genderen, C. van *et al.* Development of several organs that require inductive epithelial-mesenchymal interactions is impaired in LEF-1-deficient mice. *Genes Dev.* **8**, 2691–2703 (1994).

21. Lévy, J. *et al.* LEF1 haploinsufficiency causes ectodermal dysplasia. *Clin Genet* **97**, 595–600 (2020).

22. Deng, Q. *et al.* Deciphering gene regulatory programs in mouse embryonic skin through single-cell multiomics analysis. *Genome Biol* **26**, 187 (2025).

23. Wang, Y. *et al.* PBX1 Attenuates Hair Follicle-Derived Mesenchymal Stem Cell Senescence and Apoptosis by Alleviating Reactive Oxygen Species-Mediated DNA Damage Instead of Enhancing DNA Damage Repair. *Front Cell Dev Biol* **9**, 739868 (2021).

24. Sweeney, E., Fryer, A., Mountford, R., Green, A. & McIntosh, I. Nail patella syndrome: a review of the phenotype aided by developmental biology. *J Med Genet* **40**, 153–162 (2003).
