# Supplementary material for: Hoxc13 and homologs of mammalian hair keratins are required for the cornification of nuptial pads in Xenopus frogs
Source: Commun Biol. 2026 Jun 20;9:1302. doi: 10.1038/s42003-026-10519-y (PMC13642941; doi:10.1038/s42003-026-10519-y)
Supplement: Supplementary file 2 [file 42003_2026_10519_MOESM6_ESM.pdf]

nature portfolio

Corresponding author(s): Leopold Eckhart

Last updated by author(s): May 8, 2026

Reporting Summary

Nature Portfolio wishes to improve the reproducibility of the work that we publish. This form provides structure for consistency and transparency in reporting. For further information on Nature Portfolio policies, see our Editorial Policies and the Editorial Policy Checklist.

Statistics

For all statistical analyses, confirm that the following items are present in the figure legend, table legend, main text, or Methods section.

n/a	Confirmed	
☐	☒	The exact sample size (*n*) for each experimental group/condition, given as a discrete number and unit of measurement
☐	☒	A statement on whether measurements were taken from distinct samples or whether the same sample was measured repeatedly
☐	☒	The statistical test(s) used AND whether they are one- or two-sided<br>*Only common tests should be described solely by name; describe more complex techniques in the Methods section.*
☒	☐	A description of all covariates tested
☐	☒	A description of any assumptions or corrections, such as tests of normality and adjustment for multiple comparisons
☐	☒	A full description of the statistical parameters including central tendency (e.g. means) or other basic estimates (e.g. regression coefficient) AND variation (e.g. standard deviation) or associated estimates of uncertainty (e.g. confidence intervals)
☐	☒	For null hypothesis testing, the test statistic (e.g. *F*, *t*, *r*) with confidence intervals, effect sizes, degrees of freedom and *P* value noted<br>*Give P values as exact values whenever suitable.*
☒	☐	For Bayesian analysis, information on the choice of priors and Markov chain Monte Carlo settings
☐	☒	For hierarchical and complex designs, identification of the appropriate level for tests and full reporting of outcomes
☒	☐	Estimates of effect sizes (e.g. Cohen's *d*, Pearson's *r*), indicating how they were calculated

Our web collection on statistics for biologists contains articles on many of the points above.

Software and code

Policy information about availability of computer code

Data collection	Images were taken with an OlympusBX63 light microscope equipped with a UC-90 camera and the software cellSense Dimension (version: 2.3.18987.0,https://www.olympus-lifescience.com/en/software/cellsens/) and stereoscope with an Leica CLS 150 X external light source equipped with a Leica IC 3D camera and the software Leica Application Suite (LAS, version: 4.7.1).
Data analysis	For data analysis, we used Proteome Discoverer Software (version: 2.4.1.15) [https://www.thermofisher.com/at/en/home/industrial/mass-spectrometry/liquid-chromatography-mass-spectrometry-lc-ms/lc-ms-software/multi-omics-data-analysis/proteome-discoverer-software.html.html?erpType=Global_E1], Inkscape (versin: 1.4.2) [https://inkscape.org/release/inkscape-1.4.2/windows/64-bit/msi/?redirected=1], Graphpad Prism 8 (version 8.0.1) [https://www.graphpad.com/features], Salmon (version: 1.10.3) [https://github.com/COMBINE-lab/salmon], Python (version: 3.10) [https://www.python.org/downloads/], PyDESeq2 (version: 0.5.3) [https://pydeseq2.readthedocs.io/en/latest/], pandas (version: 2.3.2) [https://pandas.pydata.org/], numpy (version: 2.3.3) [https://numpy.org/], seaborn (version: 0.13.2) [https://seaborn.pydata.org/], matplotlib (version: 3.10.6) [https://matplotlib.org/], eggNOGmapper (version: 2) [http://eggnog-mapper.embl.de/] GOATOOLS (version: 1.5.2) [https://github.com/tanghaibao/goatools]

For manuscripts utilizing custom algorithms or software that are central to the research but not yet described in published literature, software must be made available to editors and reviewers. We strongly encourage code deposition in a community repository (e.g. GitHub). See the Nature Portfolio guidelines for submitting code & software for further information.

Data

Policy information about availability of data

All manuscripts must include a data availability statement. This statement should provide the following information, where applicable:

- Accession codes, unique identifiers, or web links for publicly available datasets
- A description of any restrictions on data availability
- For clinical datasets or third party data, please ensure that the statement adheres to our policy

Sequence data that support the findings of this study have been deposited in GenBank under the accession codes PRJNA1335124 and PRJNA1344275. The mass-spectrometry–based proteomics data have been deposited in the PRIDE repository with the project accession PXD071004. Accession codes and sources for all publicly available sequence datasets used in this study are listed in the Supplementary Materials. Gene Ontology annotations used for functional enrichment analyses were obtained from the official Gene Ontology website (http://geneontology.org/). The reference genomes used for RNA-seq analyses, X. tropicalis UCB_Xtro_10.0 (NCBI accession GCA_000004195.4) and X. laevis Xenopus_laevis_v10.1 (NCBI accession GCA_017654675.1), are available through NCBI. All data supporting the conclusions of this study are provided in the paper and/or the Supplementary materials accompany this publication.

Research involving human participants, their data, or biological material

Policy information about studies with human participants or human data. See also policy information about sex, gender (identity/presentation), and sexual orientation and race, ethnicity and racism.

Reporting on sex and gender	The research did not include human participants or human data.
Reporting on race, ethnicity, or other socially relevant groupings	The research did not include human participants or human data.
Population characteristics	The research did not include human participants or human data.
Recruitment	The research did not include human participants or human data.
Ethics oversight	The research did not include human participants or human data.

Note that full information on the approval of the study protocol must also be provided in the manuscript.

Field-specific reporting

Please select the one below that is the best fit for your research. If you are not sure, read the appropriate sections before making your selection.

☒ Life sciences ☐ Behavioural & social sciences ☐ Ecological, evolutionary & environmental sciences

For a reference copy of the document with all sections, see nature.com/documents/nr-reporting-summary-flat.pdf

Life sciences study design

All studies must disclose on these points even when the disclosure is negative.

Sample size	Sample size (n = 3) was chosen for transcriptional analysis based on the expectation that the differences in cornification between Xenopus laevis nuptial spines and control skin would be robust, similar in scale to transcriptional differences observed in strongly cornified mammalian appendages. Under this assumption, a sample size of 3 was sufficient to detect significant differences in gene expression. For proteomics, a sample size of n = 1 was used because the aim was to identify proteins present in cornified nuptial spines, rather than performing statistical comparisons. For the comparison of gene expression in forelimb skin of hoxc13-/- versus wildtype Xenopus tropicalis frogs, samples from n=2 male individuals were analyzed. The statistical analysis was performed with DESeq2, which uses the variance information across all genes to obtain stabilized variance estimates for individual genes.
Data exclusions	No data were excluded from analyses.
Replication	All experiments of quantification were performed in triplicates or quadruplicates. All attempts of replication were successful.
Randomization	Randomization was not applied because assignment to experimental groups was not applicable for this study.
Blinding	Blinding was not required because the experiments, like histological analysis, quantitative PCR, RNA-seq, and proteomics, were not sensitive to bias.

Reporting for specific materials, systems and methods

We require information from authors about some types of materials, experimental systems and methods used in many studies. Here, indicate whether each material, system or method listed is relevant to your study. If you are not sure if a list item applies to your research, read the appropriate section before selecting a response.

Materials & experimental systems

n/a	Involved in the study
☐	☒ Antibodies
☒	☐ Eukaryotic cell lines
☒	☐ Palaeontology and archaeology
☐	☒ Animals and other organisms
☒	☐ Clinical data
☒	☐ Dual use research of concern
☒	☐ Plants

Methods

n/a	Involved in the study
☒	☐ ChIP-seq
☒	☐ Flow cytometry
☒	☐ MRI-based neuroimaging

Antibodies

Antibodies used	Anti-digoxigenin antibody (Fab fragments) conjugated to alkaline phosphatase (1:5000 dilution, catalog number: 11093274910, lot number 84080920, Roche)
Validation	Anti-digoxigenin AP-conjugate was validated in published study: Komatsu Y et al. Methods Mol Biol. 2014;1092:1-15. doi: 10.1007/978-1-60327-292-6_1.

Animals and other research organisms

Policy information about studies involving animals; ARRIVE guidelines recommended for reporting animal research, and Sex and Gender in Research

Laboratory animals	This study involved Xenopus laevis frogs which were reared and bred in the animal facility of the Research Institute of Molecular Pathology (IMP), Vienna. The age of the investigated frogs was 5 years.<br>This study involved Xenopus tropicalis frogs which were reared and bred in the animal facility of the University of Gent. The age of the investigated frogs was 7 months.
Wild animals	The study did not involve wild animals.
Reporting on sex	Male and female Xenopus frogs were examined macroscopically and histologically for the presence of cornified spines on forelimb skin. In males, spines were distributed throughout the entire ventral forearm and toes, whereas in females they were restricted to the toes. Quantitative analysis of spine density revealed a higher density in males (29%) compared with females (11%).
Field-collected samples	The study does not involve samples collected from the field.
Ethics oversight	Approval of the project was obtained in a previous study (Carron et al. 2024) by the Ethical Committee for Animal Experimentation, Ghent University, Faculty of Sciences (approval number EC2023-041).<br>Wildtype Xenopus laevis frogs provided by the Elly Tanaka Lab (Research Institute of Molecular Pathology, Vienna) were killed and tissues were sampled in accordance with the guidelines of the Ethics Committee of the Medical University of Vienna.

Note that full information on the approval of the study protocol must also be provided in the manuscript.

Plants

Seed stocks	*Report on the source of all seed stocks or other plant material used. If applicable, state the seed stock centre and catalogue number. If plant specimens were collected from the field, describe the collection location, date and sampling procedures.*
Novel plant genotypes	*Describe the methods by which all novel plant genotypes were produced. This includes those generated by transgenic approaches, gene editing, chemical/radiation-based mutagenesis and hybridization. For transgenic lines, describe the transformation method, the number of independent lines analyzed and the generation upon which experiments were performed. For gene-edited lines, describe the editor used, the endogenous sequence targeted for editing, the targeting guide RNA sequence (if applicable) and how the editor was applied.*
Authentication	*Describe any authentication procedures for each seed stock used or novel genotype generated. Describe any experiments used to assess the effect of a mutation and, where applicable, how potential secondary effects (e.g. second site T-DNA insertions, mosiacism, off-target gene editing) were examined.*
