# Supplementary material for: Hoxc13 and homologs of mammalian hair keratins are required for the cornification of nuptial pads in Xenopus frogs
Source: Commun Biol. 2026 Jun 20;9:1302. doi: 10.1038/s42003-026-10519-y (PMC13642941; doi:10.1038/s42003-026-10519-y)
Supplement: Supplementary file 3 [file 42003_2026_10519_MOESM7_ESM.pdf]

nature portfolio

Peer Review File

Hoxc13 and homologs of mammalian hair keratins are required for the cornification of nuptial pads in *Xenopus* frogs

Corresponding Author: Professor Leopold Eckhart

This file contains all reviewer reports in order by version, followed by all author rebuttals in order by version.

Version 0:

Reviewer comments:

Reviewer #1

(Remarks to the Author)
In this study, the authors investigated the mechanism of the formation of the nuptial pads in Xenopus frogs. They examined transcriptomic profiles using RNA-seq analysis compared with the forelimb skin with or without a nuptial pad and found that keratin 59 and keratin 9 genes were enriched only in the nuptial pad. In addition, they analyzed the effect of the lack of the hoxc13 gene, known as a major transcription factor for direct regulation of krt59 mRNA transcription, during the formation of the nuptial pad. The authors identified that krt59 mRNA was significantly downregulated by hoxc13 knockout, and the lack of spine pigmentation and cornification. This study provides important insights into the Hoxc13 dependent differentiation of skin appendages. I consider this an important finding that bridges the gap between the keratinization of amphibian skin and the acquisition of hair in mammals within vertebrate evolution. Below are several major and minor issues for the authors to consider.

Major
1. The authors presented in situ hybridization data indicating that krt59 mRNA is expressed in keratinocytes at the tip of the spine of the nuptial pad as shown in Figure 3. However, no direct evidence has been presented that the cells expressing krt59 are keratinocytes, nor that krt59 mRNA is expressed only in this region. Therefore, the authors should explain why the krt59-expressing cells in the spine were identified as keratinocytes and provide a lower-magnification image in Figure 3.
2. The manuscript reports changes in read numbers but does not include statistical analyses. Additionally, the sample size appears to be less than three, as used in Hoxc13-deficient Xenopus. Without sufficient sample size and appropriate statistical evaluation, the reliability of the data is uncertain, and the validity of the conclusions drawn may be questionable. I recommend increasing the sample size to at least three and performing suitable statistical analyses to support the claim. Furthermore, to ensure the reliability of the RNA-seq data, validation of several key genes (e.g., keratin, hoxc12, and hoxc13) by qPCR should be performed.

Minor
1. Introduction part. Paragraph 3. To avoid readers confusion, the authors would be better to add brief explanation about frog hair keratin information if which keratin genes are classified in the hair keratin in Xenopus species in Introduction part.
2. Figure 1B, S1B and S2C-H. The scale bars should be needed.
3. P6. “Among Hox genes (Supplementary table S1), hoxc12 and hoxc13…..”. Supplemental table S1 shows expression levels of keratin genes, thus the authors should be correct the misquotation.
4. Figure 3A and C, krt59 mRNA expressed in differentiating spine cells but not in the cornified spine tips. The authors need to analyze the hoxc13 mRNA localization in the spine region by in situ hybridization, as shown in the previous report (Carron et al., 2024), to determine whether hoxc13 mRNA colocalizes with krt59 and/or krt9 mRNA.
5. P9. Discussion. Paragraph 5. The authors mentioned that only krt59 has been shown to be a direct target of Hoxc13. However, according to Figure 4C, krt9 mRNA expression is also dramatically downregulated after Hoxc13 knockout. The authors should verify whether krt59 and krt9 are direct target genes of Hoxc13 by qRT-PCR and searching the hoxc13 binding site in the krt9 promoter region.
6. The authors should clarify the mutations in the hoxc13 gene present in the knockout experiments they used.
7. According to Carron et al., 2024), Xenopus hair keratin, krt34, expressed in the claw, conserved a functional hoxc13

binding site. However, in this study, the nuptial pad lacked krt34 mRNA expression. Therefore, the authors would be better to discuss why krt34 expression was inhibited in the nuptial pad even though Hoxc13 is highly expressed in this region.

Reviewer #2

(Remarks to the Author)
This manuscript focuses on the molecular mechanisms underlying the cornification of nuptial pads in Xenopus laevis, revealing the conserved roles of the Hoxc13 transcription factor and mammalian hair keratin homologs in the formation of amphibian skin appendages. It provides critical experimental evidence for the parallel evolution of skin appendages in tetrapods. The paper features a clear logical flow and sufficient empirical support, with the generation of Hoxc13-knockout Xenopus tropicalis frogs offering compelling evidence for its conclusions. While the study is highly interesting, I have a few minor questions and suggestions for the authors to address, and I recommend the acceptance of this manuscript.

Specific questions and suggestions are as follows:

1.The authors state that nuptial pads are present in both female and male Xenopus laevis, citing a 1981 reference, yet this is inconsistent with my current understanding. The authors are advised to consult a broader range of references to clarify this claim. Cornified spines are found on the forelimbs of both female and male frogs, but it seems that only the cornified spines covering the entire ventral surface of the forelimbs are defined as nuptial pads. My understanding may be inaccurate, and I hope the authors can clarify this point.
2. Given the significant sexual dimorphism of cornified spines on the forelimbs of female and male frogs, it would be preferable for Figure 4 to specify the sex and age of both wild-type and knockout frogs. I assume the comparison was conducted between sexually mature male frogs, and this should be explicitly stated.
3. I am curious to know whether Hoxc13-knockout frogs exhibit morphological differences in other tissues and structures besides nuptial pads. For instance, do claws still develop? Are there any other changes in the skeletal structure or skin of the forelimbs?
4. In the discussion section accompanying Figure 5, the authors are suggested to include humans as a representative species. Additionally, it would be worthwhile to address the following questions: Is the hoxc13 gene lost in snakes? The collinearity of Hox genes is relatively conserved in amphibians, but the authors do not discuss the status of hoxc13 in birds. Avian evolution has been accompanied by extensive gene loss—does hoxc13 persist in birds, and is this gene associated with feather formation? What is the expression pattern of hoxc13 in fish, which are phylogenetically basal to amphibians? As this is the discussion section, the authors are not required to provide definitive answers to all these questions.

Version 1:

Reviewer comments:

Reviewer #1

(Remarks to the Author)
The authors have carefully addressed all of my comments and made appropriate revisions in this revision round. No further revisions are necessary, and the manuscript is recommended for acceptance.
Although this is beyond the scope of the present paper, the authors consider that the differences in the transcriptional regulation of the krt34 and krt59 genes in claws and nuptial pads by Hoxc13 will be an important point to consider regarding the molecular evolution of hair keratin genes in the future work.

Reviewer #2

(Remarks to the Author)
All my questions have been well answered by the authors. I recommend accepting this manuscript.

Open Access This Peer Review File is licensed under a Creative Commons Attribution 4.0 International License, which permits use, sharing, adaptation, distribution and reproduction in any medium or format, as long as you give appropriate credit to the original author(s) and the source, provide a link to the Creative Commons license, and indicate if changes were made.
In cases where reviewers are anonymous, credit should be given to 'Anonymous Referee' and the source.
The images or other third party material in this Peer Review File are included in the article's Creative Commons license, unless indicated otherwise in a credit line to the material. If material is not included in the article's Creative Commons license and your intended use is not permitted by statutory regulation or exceeds the permitted use, you will need to obtain permission directly from the copyright holder.
To view a copy of this license, visit https://creativecommons.org/licenses/by/4.0/

Response to the reviewers' comments

Reviewer #1 (Remarks to the Author):

In this study, the authors investigated the mechanism of the formation of the nuptial pads in Xenopus frogs. They examined transcriptomic profiles using RNA-seq analysis compared with the forelimb skin with or without a nuptial pad and found that keratin 59 and keratin 9 genes were enriched only in the nuptial pad. In addition, they analyzed the effect of the lack of the hoxc13 gene, known as a major transcription factor for direct regulation of krt59 mRNA transcription, during the formation of the nuptial pad. The authors identified that krt59 mRNA was significantly downregulated by hoxc13 knockout, and the lack of spine pigmentation and cornification. This study provides important insights into the Hoxc13 dependent differentiation of skin appendages. I consider this an important finding that bridges the gap between the keratinization of amphibian skin and the acquisition of hair in mammals within vertebrate evolution. Below are several major and minor issues for the authors to consider.

<u>Response:</u>
We thank the reviewer for the positive comments and constructive suggestions.

<u>Major</u>
1. The authors presented in situ hybridization data indicating that krt59 mRNA is expressed in keratinocytes at the tip of the spine of the nuptial pad as shown in Figure 3. However, no direct evidence has been presented that the cells expressing krt59 are keratinocytes, nor that krt59 mRNA is expressed only in this region. Therefore, the authors should explain why the krt59-expressing cells in the spine were identified as keratinocytes and provide a lower-magnification image in Figure 3.

<u>Response</u>:
In the revised manuscript, we emphasize that the spines of the nuptial pads are epithelial structures that consist, with the exception of very sparsely distributed Langerhans cells and dendritic cells, of keratinocytes. Studies by Kurabuchi and others, cited in our manuscript, have shown the epithelial cell layers that correspond to stages of keratinocyte differentiation. We hope that the revised text (especially the second paragraph of the Introduction), additional labeling of the epidermal compartment in Figure 1C and Supplementary Figure S1C and D, and the diagram shown in Figure 1E, clarify that the krt59-expressing cells can only be keratinocytes. As suggested by the reviewer, we provide lower-magnification images in Figure 3.

2. The manuscript reports changes in read numbers but does not include statistical analyses. Additionally, the sample size appears to be less than three, as used in Hoxc13-deficient Xenopus. Without sufficient sample size and appropriate statistical evaluation, the reliability of the data is uncertain, and the validity of the conclusions drawn may be questionable. I recommend increasing the sample size to at least three and performing suitable statistical analyses to support the claim. Furthermore, to ensure the reliability of the RNA-seq data, validation of several key genes (e.g., keratin, hoxc12, and hoxc13) by qPCR should be performed.

<u>Response</u>:
We appreciate the comment and the recommendation of the reviewer. We have included a statistical analysis for the experiment, which was missing in the original version of the manuscript. While the comparison of gene expression in the nuptial pads versus the skin of *X. laevis* utilized three biological replicates (Figure 2 and Tables 1 and 2), as suggested by the reviewer, the comparative analysis of WT vs. Hoxc13-deficient *X. tropicalis* frogs could only be performed with two frogs per group (Figure 4). Unfortunately, larger numbers of Hoxc13-knockout animals are not available and will not be available within the next months.

To address the comment of the reviewer, we provide the statistical analysis performed with the DESeq2 software package, which allows the statistical analysis of high-throughput sequencing data with small numbers of samples. The P-value was calculated based on estimates of variance. Variance estimation use the variance information across all genes to estimate variance for individual genes. This allows the calculation of a P-value and a Benjamini Hochberg-corrected adjusted P-value. As this estimation is reliable for genes with high read counts in at least one group (WT or KO), we show only genes with a normalized read count >20000 in wildtype (wt) tissue (Figure 4C and Supplementary Table S1). Only the expression level differences of genes krt59, krt50, krt78.6, krt61, krt17, and krt9.1 have a Padj<0.05, whereby – due to extremely high fold change and low variance – the Padj=1.03E-74 for the hair keratin homolog krt59 is extremely small. The following Figure for the reviewer illustrates the small variance of data for the biological replicates of wildtype (WT, upper panel) and Hoxc13 knockout (lower panel) forelimb toe skin, and the impact of Hoxc13 knockout (lower panel) on the expression level of *krt59*.

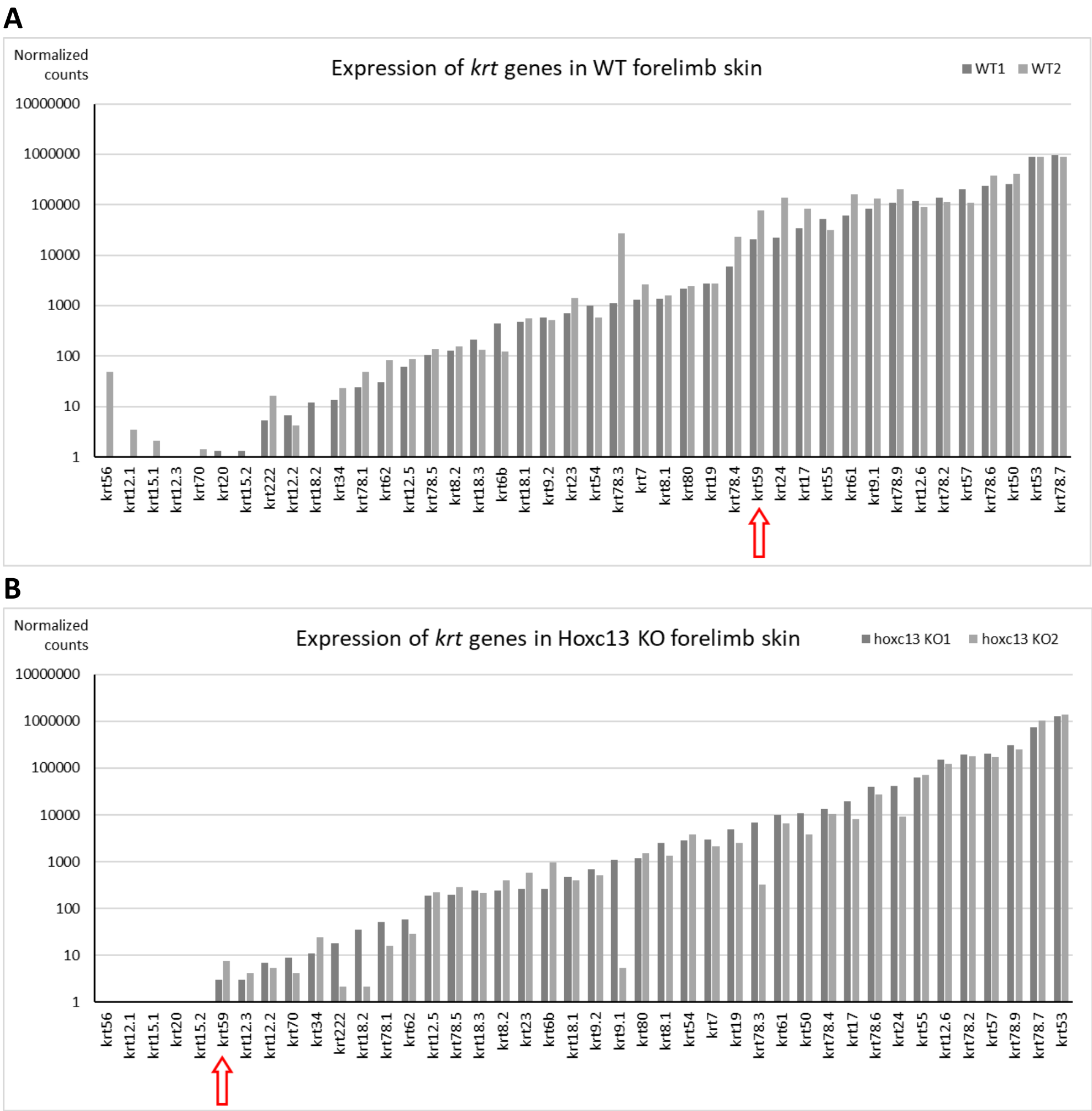

Figure for the reviewer: Expression levels of *krt* genes in wildtype and Hoxc13 KO forelimb toe skin. The normalized counts of RNA-seq reads of *krt* genes are shown as bars for each frog. Panel A shows the values of wildtype frogs (WT1, WT2), and panel B shows the values of Hoxc13 KO frogs (KO1, KO2). Note that the normalized counts are shown at a logarithmic scale (vertical axis). The *krt* genes (horizontal axis) are sorted according to normalized read counts in WT1 (A) and KO1 (B). The position of the hair keratin homolog *krt59* is marked by a red arrow.

<u>Minor</u>

1. Introduction part. Paragraph 3. To avoid readers confusion, the authors would be better to add brief explanation about frog hair keratin information if which keratin genes are classified in the hair keratin in Xenopus species in Introduction part.

Response:
To address the comment of the reviewer, we have added a sentence explaining the expression of hair keratin homologs Krt34 (type I) and Krt59 (type II) in *Xenopus* frogs in the third paragraph of the Introduction: “In *Xenopus* frogs, the hair keratin homologs Krt34 (type I) and Krt59 (type II) are expressed in the cornified claws (Carron et al. 2024), which is equivalent to the co-expression of type I and type II hair keratins in the claws of anole lizards (Eckhart et al. 2008).”

2. Figure 1B, S1B and S2C-H. The scale bars should be needed.

Response:
The scale bars have been added.

3. P6. “Among Hox genes (Supplementary table S1), hoxc12 and hoxc13…..”. Supplemental table S1 shows expression levels of keratin genes, thus the authors should be correct the misquotation.

Response:
We have deleted “(Supplementary table S1)” in this sentence.

4. Figure 3A and C, krt59 mRNA expressed in differentiating spine cells but not in the cornified spine tips. The authors need to analyze the hoxc13 mRNA localization in the spine region by in situ hybridization, as shown in the previous report (Carron et al., 2024), to determine whether hoxc13 mRNA colocalizes with krt59 and/or krt9 mRNA.

Response:
As suggested by the reviewer, we have performed mRNA *in situ* hybridization experiments for *hoxc13* in nuptial pads. In contrast to the claw epithelium, the epithelial cells of the nuptial pads did not yield a signal, suggesting that the expression level *hoxc13* in nuptial pads is below the detection limit of the method and lower than in claws. Together with the finding that *krt34*, the type I hair keratin homolog that depends on Hoxc13 for expression in *Xenopus* claws (Carron et al. 2024), is not expressed in nuptial pads, we conclude that the role of Hoxc13 in controlling keratin expression is different in the nuptial pads than in claws. The third paragraph of the Discussion has been changed accordingly by adding the following text:
“However, the absence of expression of *krt34*, which like *krt59* contains a Hoxc13 binding site in its proximal promoter (Carron et al. 2024), suggests that keratin expression is not controlled by Hoxc13 alone. The reason for the difference between *krt34* and *krt59* expression patterns is currently unknown but may involve differential dependency on additional transcriptional activators besides Hoxc13, active repression of *krt34* in the nuptial pads, or the dependence of *krt34* on higher levels of Hoxc13 than those present in nuptial pad epithelial cells. Although abundant according to the RNA-seq analysis, *hoxc13* mRNA was not detectable by *in situ* hybridization in nuptial pads. By contrast, the claw epithelium was clearly positive in *hoxc13 in situ* hybridization (Carron et al. 2024), suggesting a lower level of expression of *hoxc13* in nuptial pads than in the claws.“

5. P9. Discussion. Paragraph 5. The authors mentioned that only krt59 has been shown to be a direct target of Hoxc13. However, according to Figure 4C, krt9 mRNA expression is also dramatically downregulated after Hoxc13 knockout. The authors should verify whether krt59 and krt9 are direct target genes of Hoxc13 by qRT-PCR and searching the hoxc13 binding site in the krt9 promoter region.

Response:
As we have discussed in response to the reviewer’s point 4, we conclude that “keratin expression is not controlled by Hoxc13 alone in nuptial pads”. As suggested by the reviewer, we analyzed the promoter sequences of *krt9* genes in *X. laevis* and *X. tropicalis* and found a potential binding site

(ATTATAGA) at position -149 relative to the TATA box of *X. tropicalis krt9* and at the corresponding position of *X. laevis krt9* homeologs. However, this sequence deviates from the consensus motif at position 1 (A instead of T), and therefore its functionality is uncertain. Importantly, the statistical analysis shows that the significance of the downregulation of krt9.1 is only borderline (Padj = 0.0313). Therefore, we do not specifically discuss a regulation of *krt9.1* by Hoxc13 in the revised manuscript, but rather make a general statement on keratins in the first sentence to the fourth paragraph of the Discussion, which was changed to "Besides *krt59*, other members of the large family of keratin genes (Nishikawa et al. 1992) were also affected either directly or indirectly by the loss of Hoxc13 (Figure 4C, Supplementary Tables S4 and S5)."

6. The authors should clarify the mutations in the hoxc13 gene present in the knockout experiments they used.

<u>Response</u>:
We provide the information about hoxc13 mutations in the new Supplementary Figure S4 of the revised manuscript.

7. According to Carron et al., 2024), Xenopus hair keratin, krt34, expressed in the claw, conserved a functional hoxc13 binding site. However, in this study, the nuptial pad lacked krt34 mRNA expression. Therefore, the authors would be better to discuss why krt34 expression was inhibited in the nuptial pad even though Hoxc13 is highly expressed in this region.

<u>Response</u>:
We agree with the reviewer that the lack of *krt34* expression in the nuptial pad is surprising. This result was obtained in both transcriptome (Table 1) and proteome (Suppl. Data S2) studies of *X. laevis* and transcriptome studies of *X. tropicalis* (Suppl. Table S2, where the number of RNA-seq read counts of *krt34* was more than 1000-fold less than those of krt59). As suggested by the reviewer, we discuss this unexpected finding and the potential differences in the regulation of *krt34* and *krt59* in the third paragraph of the Discussion of the revised manuscript. Please see our response to comment 4.

<u>Reviewer #2 (Remarks to the Author):</u>

This manuscript focuses on the molecular mechanisms underlying the cornification of nuptial pads in Xenopus laevis, revealing the conserved roles of the Hoxc13 transcription factor and mammalian hair keratin homologs in the formation of amphibian skin appendages. It provides critical experimental evidence for the parallel evolution of skin appendages in tetrapods. The paper features a clear logical flow and sufficient empirical support, with the generation of Hoxc13-knockout Xenopus tropicalis frogs offering compelling evidence for its conclusions. While the study is highly interesting, I have a few minor questions and suggestions for the authors to address, and I recommend the acceptance of this manuscript.

<u>Response:</u>
We thank the reviewer for the positive comments and constructive suggestions.

Specific questions and suggestions are as follows:

1.The authors state that nuptial pads are present in both female and male Xenopus laevis, citing a 1981 reference, yet this is inconsistent with my current understanding. The authors are advised to consult a broader range of references to clarify this claim. Cornified spines are found on the forelimbs of both female and male frogs, but it seems that only the cornified spines covering the entire ventral surface of the forelimbs are defined as nuptial pads. My understanding may be inaccurate, and I hope the authors can clarify this point.

Response:
As suggested by the reviewer, we have added further information on this point and more citations in the Introduction. Several studies have identified cornified epidermal projections in species of anurans, but they are not associated with “sexually dimorphic skin glands”, which are considered a functionally important components of nuptial pads in male anurans (Luna et al. 2018). Therefore, we have changed the terminology in the revised manuscript and, instead of “nuptial pads”, we use the term “nuptial pad-like structures” for samples from female frogs. This follows the suggestion of Jeckel et al. 2019.

2. Given the significant sexual dimorphism of cornified spines on the forelimbs of female and male frogs, it would be preferable for Figure 4 to specify the sex and age of both wild-type and knockout frogs. I assume the comparison was conducted between sexually mature male frogs, and this should be explicitly stated.

Response:
Yes, the comparison was conducted between sexually mature male frogs. This is explicitly stated in the legend of the revised figure.

3. I am curious to know whether Hoxc13-knockout frogs exhibit morphological differences in other tissues and structures besides nuptial pads. For instance, do claws still develop? Are there any other changes in the skeletal structure or skin of the forelimbs?

Response:
The cornified claws do not develop on the hindlimbs of Hoxc13 knockout frogs. We have reported this phenotype (Carron et al. 2024). The skin of the forelimbs did not show any other obvious changes than those of the cornified epithelial spines. However, we have not investigated in detail other traits such as the morphology and density of glands. To address this point, we have added the following sentence at the end of the last-but-one paragraph of the Discussion: “While the present study focused on the cornified epithelial spines of nuptial pads, the abundance and differentiation of skin glands, which are also important for the function of nuptial pads (Luna et al. 2018), remain to be investigated with regard to their keratin profile and their dependence on Hoxc13.”

Skeletal structures were not specifically investigated in this study but did not appear to be different. The role of hoxc13 in skeletal development was investigated in a recent study, which however did not show effects of hoxc13 knockout on the limbs of frogs (Kawasumi-Kita et al. hoxc12/c13 as key regulators for rebooting the developmental program in *Xenopus* limb regeneration. Nat Commun. 2024;15:3340).

4. In the discussion section accompanying Figure 5, the authors are suggested to include humans as a representative species. Additionally, it would be worthwhile to address the following questions: Is the hoxc13 gene lost in snakes? The collinearity of Hox genes is relatively conserved in amphibians, but the authors do not discuss the status of hoxc13 in birds. Avian evolution has been accompanied by extensive gene loss—does hoxc13 persist in birds, and is this gene associated with feather formation? What is the expression pattern of hoxc13 in fish, which are phylogenetically basal to amphibians? As this is the discussion section, the authors are not required to provide definitive answers to all these questions.

Response:
We thank the reviewer for these suggestions, which we have considered carefully. In Figure 5, we show the mouse to make this figure easily comparable to the summary figure of our previous study on hair keratins and Hoxc13 in claws (Carron et al. 2024).
As mentioned in our recent paper (Carron et al. 2024), hoxc13 is expressed in the tail during

development of all vertebrates including fish and presumably also snakes. The hoxc13 gene is conserved in snakes and birds, as it has been published previously (Liang et al. A general scenario of Hox gene inventory variation among major sarcopterygian lineages. BMC Evol Biol. 2011;11:25). Chicken hoxc13 is expressed in the embryonic tail (Li et al. Skin regional specification and higher-order HoxC regulation. Sci Adv. 2025;11:eado2223) and in the tips of digits that develop claws, but not in wing digits that do not develop claws (Fernandez-Guerrero et al. Mammalian-specific ectodermal enhancers control the expression of Hoxc genes in developing nails and hair follicles. Proc Natl Acad Sci U S A. 2020;117:30509-30519). By mRNA in situ hybridization, hoxc13 was also not detected in developing feathers (Fernandez-Guerrero et al. Mammalian-specific ectodermal enhancers control the expression of Hoxc genes in developing nails and hair follicles. Proc Natl Acad Sci U S A. 2020;117:30509-30519).

Most of these points were discussed in our recent paper (Carron et al. 2024). To partly address the comment of the reviewer, we have added the following sentence in the fourth paragraph of the Introduction: "In the chicken, Hoxc13 is expressed in developing claws (Fernandez-Guerrero et al. 2020)." The conservation of only one hair keratin homolog in birds is mentioned at the end of the second paragraph of the Discussion.

Other changes made in the course of the revision of the manuscript

We have changed the format (capital letters) of several labels in Figure 5. For clarification of the focus of this figure, we have changed the wording over the blue arrow over the root of the simplified phylogenetic tree of tetrapods. Instead of "Origin of hair keratin orthologs", we write "Origin of Hoxc13-regulated hair keratins".

In the second sentence of the third paragraph of the Discussion, we have deleted "tail scales" from the list of defective structures in Hoxc13 knockout mice, because they were actually reported to be "unaffected under gross observation" (Godwin and Capecchi 1998).

We have added the following references:

Anders S, Huber W. Differential expression analysis for sequence count data. Genome Biol. 2010;11:R106. doi: 10.1186/gb-2010-11-10-r106.
Fox H. The skin of Amphibia. In: Biology of the integument. Vol 2. Vertebrates (eds. Bereiter-Hahn J, Matoltsy AG, Richards KS) 78–135 (Springer 1986).
Jeckel A, Caorsi V, Grant T, Borges-Martins M. The Nuptial Pads of Melanophryniscus (Anura: Bufonidae), with the Unexpected Occurrence of Nuptial-Pad–Like Structures in Females of Two Species. J Herpetol 2019;53:53–61. doi: 10.1670/18-104.
Kurabuchi S. Fine structure of nuptial pad surface of male ranid frogs. Tissue Cell. 1993;25:589-98. doi: 10.1016/0040-8166(93)90011-9.
Love MI, Huber W, Anders S. Moderated estimation of fold change and dispersion for RNA-seq data with DESeq2. Genome Biol. 2014;15:550. doi: 10.1186/s13059-014-0550-8.
Orton F, Svanholm S, Jansson E, Carlsson Y, Eriksson A, Uren Webster T, McMillan T, Leishman M, Verbruggen B, Economou T, Tyler CR, Berg C. A laboratory investigation into features of morphology and physiology for their potential to predict reproductive success in male frogs. PLoS One 2020;15:e0241625. doi: 10.1371/journal.pone.0241625.
Tang Z, Chen Y, Ren B, Wang X, Zhang H, Han Y, Yuan Z, Weng Q. Immunoreactivities of AR, ERα, ERβ and aromatase in the nuptial pad of Chinese brown frog (Rana dybowskii) during pre-hibernation and the breeding period. Eur J Histochem. 2021;65:3206. doi: 10.4081/ejh.2021.3206.
Varga JFA, Bui-Marinos MP, Katzenback BA. Frog Skin Innate Immune Defences: Sensing and Surviving Pathogens. Front Immunol. 2019;9:3128. doi: 10.3389/fimmu.2018.03128.

Several typos were corrected.
