# Supplementary material for: Hoxc13 and homologs of mammalian hair keratins are required for the cornification of nuptial pads in Xenopus frogs
Source: Commun Biol. 2026 Jun 20;9:1302. doi: 10.1038/s42003-026-10519-y (PMC13642941; doi:10.1038/s42003-026-10519-y)
Supplement: Supplementary file 4 [file 42003_2026_10519_MOESM2_ESM.docx]

**Description of Additional Supplementary Files**

File name: Supplementary Data S1

Description: RNA-seq analysis of Xenopus laevis forelimb skin with nuptial pads versus control hindlimb skin

File name: Supplementary Data S2

Description: Proteomics of Xenopus laevis forelimb skin with nuptial pads

File name: Supplementary Data S3

Description: Source data of the graphs in Figure 2, Figure 4C and Supplementary Figure S2I
